# Identification of Type 1 Diabetes–Associated DNA Methylation Variable Positions That Precede Disease Diagnosis

**DOI:** 10.1371/journal.pgen.1002300

**Published:** 2011-09-29

**Authors:** Vardhman K. Rakyan, Huriya Beyan, Thomas A. Down, Mohammed I. Hawa, Siarhei Maslau, Deeqo Aden, Antoine Daunay, Florence Busato, Charles A. Mein, Burkhard Manfras, Kerith-Rae M. Dias, Christopher G. Bell, Jörg Tost, Bernhard O. Boehm, Stephan Beck, R. David Leslie

**Affiliations:** 1 Blizard Institute of Cell and Molecular Science, Barts and The London School of Medicine and Dentistry, Queen Mary University of London, London, United Kingdom; 2 The Gurdon Institute and Department of Genetics, University of Cambridge, Cambridge, United Kingdom; 3 Laboratory for Functional Genomics, Fondation Jean-Dausset – CEPH, Paris, France; 4 Laboratory for Epigenetics, Centre National de Génotypage, CEA – Institut de Génomique, Evry, France; 5 The Genome Centre, Queen Mary University of London, Barts and The London School of Medicine and Dentistry, The Sir John Vane Science Centre, London, United Kingdom; 6 Division of Endocrinology and Diabetes, Department of Internal Medicine I, University Medical Center Ulm and Center of Excellence “Metabolic Disorders” Baden-Württemberg, Ulm, Germany; 7 UCL Cancer Institute, University College London, London, United Kingdom; University of Liège, Belgium

## Abstract

Monozygotic (MZ) twin pair discordance for childhood-onset Type 1 Diabetes (T1D) is ∼50%, implicating roles for genetic and non-genetic factors in the aetiology of this complex autoimmune disease. Although significant progress has been made in elucidating the genetics of T1D in recent years, the non-genetic component has remained poorly defined. We hypothesized that epigenetic variation could underlie some of the non-genetic component of T1D aetiology and, thus, performed an epigenome-wide association study (EWAS) for this disease. We generated genome-wide DNA methylation profiles of purified CD14^+^ monocytes (an immune effector cell type relevant to T1D pathogenesis) from 15 T1D–discordant MZ twin pairs. This identified 132 different CpG sites at which the direction of the intra-MZ pair DNA methylation difference significantly correlated with the diabetic state, i.e. T1D–associated methylation variable positions (T1D–MVPs). We confirmed these T1D–MVPs display statistically significant intra-MZ pair DNA methylation differences in the expected direction in an independent set of T1D–discordant MZ pairs (P = 0.035). Then, to establish the temporal origins of the T1D–MVPs, we generated two further genome-wide datasets and established that, when compared with controls, T1D–MVPs are enriched in singletons both before (P = 0.001) and at (P = 0.015) disease diagnosis, and also in singletons positive for diabetes-associated autoantibodies but disease-free even after 12 years follow-up (P = 0.0023). Combined, these results suggest that T1D–MVPs arise very early in the etiological process that leads to overt T1D. Our EWAS of T1D represents an important contribution toward understanding the etiological role of epigenetic variation in type 1 diabetes, and it is also the first systematic analysis of the temporal origins of disease-associated epigenetic variation for any human complex disease.

## Author Summary

Type 1 diabetes (T1D) is a complex autoimmune disease affecting >30 million people worldwide. It is caused by a combination of genetic and non-genetic factors, leading to destruction of insulin-secreting cells. Although significant progress has recently been made in elucidating the genetics of T1D, the non-genetic component has remained poorly defined. Epigenetic modifications, such as methylation of DNA, are indispensable for genomic processes such as transcriptional regulation and are frequently perturbed in human disease. We therefore hypothesized that epigenetic variation could underlie some of the non-genetic component of T1D aetiology, and we performed a genome-wide DNA methylation analysis of a specific subset of immune cells (monocytes) from monozygotic twins discordant for T1D. This revealed the presence of T1D–specific methylation variable positions (T1D–MVPs) in the T1D–affected co-twins. Since these T1D–MVPs were found in MZ twins, they cannot be due to genetic differences. Additional experiments revealed that some of these T1D–MVPs are found in individuals before T1D diagnosis, suggesting they arise very early in the process that leads to overt T1D and are not simply due to post-disease associated factors (e.g. medication or long-term metabolic changes). T1D–MVPs may thus potentially represent a previously unappreciated, and important, component of type 1 diabetes risk.

## Introduction

Type 1 diabetes (T1D) is a complex autoimmune disease affecting more than 30 million people worldwide [1]. It is caused by a combination of genetic and non-genetic factors [1]–[3], leading to immune destruction of insulin-secreting islet cells. A role for non-genetic factors is suggested by studies of migrant populations, the recent rise in T1D prevalence, and twin-cohorts [3], [4]. For example, a monozygotic (MZ) twin of a T1D–affected co-twin will not always develop the disease, only ∼50% do so, even though MZ twins are genetically identical [3], [5], [6]. It has been proposed that these non-genetic factors could take the form of environmental influences such as viral infections, dietary factors, or vitamin D deficiency [7]. However, none of these have been conclusively proven to play a role in T1D etiology and compared with the recent progress in elucidating T1D–associated genetic variants, non-genetic factors have remained poorly defined.

We therefore hypothesized that epigenetic variation contributes to the non-genetic component of T1D etiology. Epigenetic modifications, such as DNA methylation and post-translational histone modifications, are indispensable for a variety of genomic processes including transcriptional regulation and maintenance of genomic integrity [8]. Their importance is further highlighted by the association between epigenetic perturbations and cancer [8]. More recently, and relevant to our hypothesis that T1D aetiopathogenesis has an epigenetic component, it has been found that epigenetic perturbations are also associated with non-malignant diseases, including autoimmune conditions, and that MZ twins can be epigenetically discordant i.e. epigenetic variation can exist in the absence of genetic heterogeneity [9]–[15]. We therefore decided to perform an epigenome-wide association study (EWAS) to identify epigenetic variation that, in combination with genes and environment, could alter T1D susceptibility by potentially influencing the functions of key immune effector cells, given that the majority of known T1D–associated genetic variants are in, or near, genes that predominantly function in such cell types [5]. Importantly, we wanted to rule out genetic differences as the basis of any identified T1D–associated epigenetic variation, and also better understand whether such epigenetic variants are potentially causal for, or consequential to, the disease process. Making these distinctions is critical for subsequent elucidation of the etiological role of disease-associated epigenetic variation. We therefore devised a novel EWAS strategy that combines T1D–discordant MZ twins with longitudinally sampled pre–T1D singletons to rule out genetic differences and establish the temporal origins of T1D–associated epigenetic variation.

## Results

### Identification of T1D–associated DNA methylation variable positions (T1D–MVPs)

For the initial genome-wide screen, we recruited 15 T1D–discordant MZ twin pairs (Table S1), selected according to the following criteria: (i) European origin; (ii) both co-twins available for study; (iii) diabetic twin had T1D diagnosed at <20 years of age; (iv) neither twin was receiving drugs other than human insulin for the diabetic twin; (v) diabetic twin had no current major diabetes complications; (vi) non-diabetic twin had low disease risk, that is <2% based on the lack of diabetes-associated autoantibodies (GADA, I-A2A and ZnT8A), and normal glucose tolerance [5], [16]. From these twins, we isolated CD14^+^ monocytes for subsequent DNA methylation analysis. Monocytes are immune effector cells that give rise to tissue macrophages that have been associated with the destruction of the islet cells, causing insulin deficiency [17]–[19]. Furthermore, monocytes can be obtained to >90% purity (Figure S1), thereby minimizing detection of apparent epigenetic changes due to altered proportions of cell subtypes (as might happen with whole blood), and loss of sensitivity due to tissue-specificity of inter-individual epigenetic variation. Finally, monocytes have a short lifespan of a few weeks, so are less likely to harbor post-differentiation, random epigenetic alterations. DNA methylation profiling was performed using Illumina HumanMethylation27 BeadChips (Illumina 27K) [20], that allow genome-wide single-CpG resolution DNA methylation measurements at 27,458 different CpG sites within 14,475 promoters (per promoter there are approximately 2 CpG sites usually spaced between 500–2,000 bp apart), and correlate well with bisulfite PCR sequencing (R^2^ = 0.88, Figure S2).

**Figure 1:**
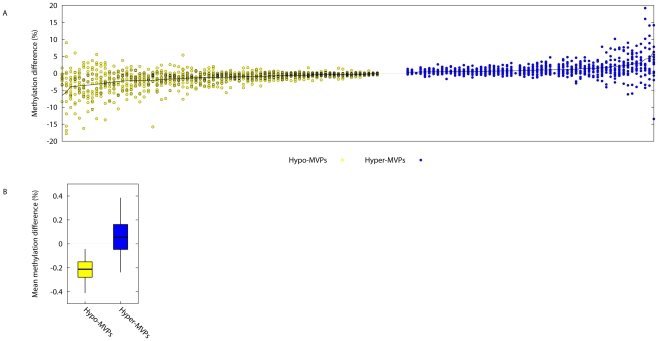
Identification of Type 1 Diabetes-associated DNA methylation variable positions (T1D–MVPs). (A) On the x-axis, each ‘column’ of data contains 15 different points, indicating the absolute intra-pair DNA methylation difference observed at a single T1D–MVP for each of the 15 different T1D–discordant MZ pairs. Plotted are the 58 different hyper- and 74 different hypoT1D–MVPs that were called at P<0.01. Data are arranged in order of decreasing or increasing absolute intra-MZ pair methylation differences for hypo- (yellow) and hyper- (blue) T1D–MVPs respectively (Refer to Table S2 for numerical values). (B) For each T1D–MVP, we asked whether experimental data is available for a neighboring CpG site within 2 kb genomic distance. In cases where several such CpG sites were present, we considered only the closest neighbor. These data were available for 56 Hypo-T1D–MVPs (out of total 74) and for 39 Hyper-T1D–MVPs (out of total 58). We then quantified the T1D–specific methylation change of these neighboring CpG sites, and plotted the intervals, such that boxes cover 50% and whiskers 95% of the data range.

Following array Q.C. and normalization, the final dataset comprised of 22,645 (of the total 27,458) CpG sites (Materials and Methods). We used Wilcoxon signed rank tests to identify T1D–associated intra-pair DNA methylation differences at each CpG site i.e. T1D–associated methylation variable positions (T1D–MVPs). This statistical procedure tests the null hypothesis that there is no intra-twin pair difference in methylation, and also takes into account the arrangement of genetically identical T1D–affected and healthy individuals into T1D–discordant MZ twin pairs. At P<0.01 – a pragmatic threshold for selecting CpG sites for further study – we identified 58 T1D–MVPs hypermethylated (hyperT1D–MVPs), and 74 T1D–MVPs hypomethylated (hypoT1D–MVPs) in the T1D–affected co-twins (Table S2). The number of MVPs observed at this threshold was significantly higher than would be expected by chance (P = 0.02). Mean intra-pair differences in DNA methylation levels at T1D–MVPs ranged from 0.13%–6.6% (Figure 1A and Table S2), in line with recent findings that inter-individual epigenetic variation in the context of human non-malignant complex diseases and phenotypes is almost invariably of small magnitude [9]–[15].

Examination of CpGs neighboring the ‘index’ T1D–MVPs revealed that they showed similar directional DNA methylation differences, although just short of significance (P = 0.06, Figure 1B). The power to perform this type of analysis using the Illumina27K platform is somewhat limited since the spacing between the two different CpG sites per promoter, in the majority of cases, is greater than the 500 bp range over which correlation of DNA methylation has previously been reported to decay [21]. Therefore, although not conclusive, our analysis suggests that at least T1D–MVPs are likely to be within larger T1D–associated differentially methylated regions (DMRs).

### T1D–MVPs display decreased intra-pair variability in control MZ pairs

**Figure 2:**
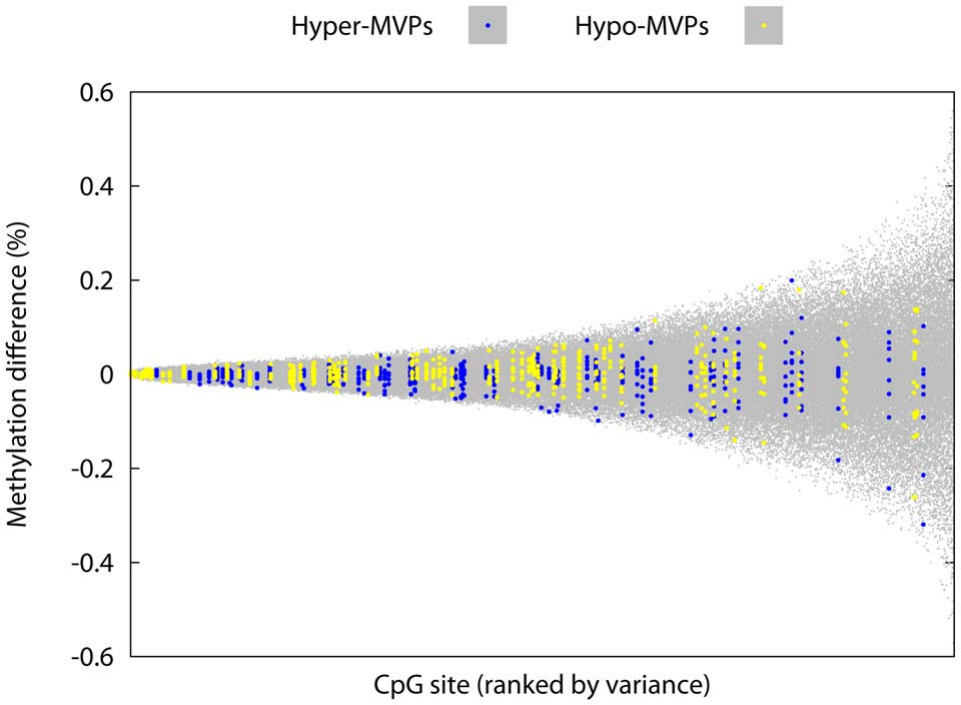
T1D–MVPs are not due to increased technical and/or biological variability. We generated Illumina27K profiles for CD14^+^ cells from 9 different control MZ pairs and calculated intra-MZ pair methylation differences at each probe on the Illumina27K array, for each of the 9 different MZ pairs. Here we plot the variance (around the mean) observed in intra-MZ pair methylation differences at each probe over all 9 MZ pairs (i.e. each ‘column’ of data contains 9 different data-points). For each MZ pair, the choice of the ‘index’ co-twin was arbitrary. CpG sites were ranked in order of increasing sample variance across the 9 intra-pair differences measured at each site. The range of intra-MZ pair variability at T1D–MVP-corresponding probes (highlighted) is significantly less compared with the range of variability observed across other probes on the array (P = 2.4×10^−8^, Welch's t-test). Number of probes used in this analysis = 22,645 (of the total 27,458 probes on the array. Refer to the section ‘Array Processing’ in Materials and Methods for the Q.C. steps performed on the arrays).

As with any microarray-based platform, different probes on the Illumina27K array are associated with inherently different levels of technical variability, or experimental noise. Additionally, it is known that some CpG sites in the human genome are intrinsically epigenetically metastable, that is, they display elevated levels of inter-individual variation that is not explained by genetic heterogeneity [11]. To test whether these sources of technical or biological ‘noise’ potentially influence our results, we generated Illumina27K profiles for CD14^+^ cells from 9 different control MZ pairs (i.e. both co-twins are T1D–unaffected). We calculated intra-MZ pair methylation differences at each probe on the Illumina27K array, for each of the 9 different MZ pairs, and then calculated the variance observed in intra-MZ pair methylation differences at each probe over all 9 MZ pairs (Figure 2). We found that the range of intra-MZ pair variability at T1D–MVP-corresponding probes is significantly less compared with the range of variability observed across other probes in the control MZ pairs (P = 2.4×10^−8^, Welch's t-test). Although this analysis does not distinguish between technical and biological variance, it does strongly suggest that the measured intra-MZ pair methylation differences at T1D–MVPs are not simply the result of higher-than-average levels of technical or biological noise.

### Pyrosequencing-based analysis and independent biological confirmation of T1D–MVPs

We then attempted to confirm the T1D–MVPs using two approaches: pyrosequencing-based bisulfite PCR analysis, and also array-based biological confirmation in an independent set of T1D–discordant twins. For the pyrosequencing, we randomly chose 24 different T1D–MVPs – 16 hyperT1D–MVPs and 8 hypoT1D–MVPs. These were assayed in CD14^+^ cells from each of 15 different T1D–discordant MZ pairs (10 from the original 15 used in the Illumina27K screen, and 5 additional pairs), i.e. a total 720 different pyrosequencing reactions performed according to standard procedures (Materials and Methods). We obtained complete data for all samples for 13 amplicons corresponding to 5 hyper-MVPs and 8 hypo-MVPs (all raw data are shown in Table S3). A group-wise analysis of these 13 amplicons revealed that hyperT1D–MVPs displayed an overall trend towards greater methylation levels in the affected co-twins compared with hypoT1D–MVPs, although this difference was just short of significance (P = 0.063, Welch's t-test, Figure S3).

**Figure 3:**
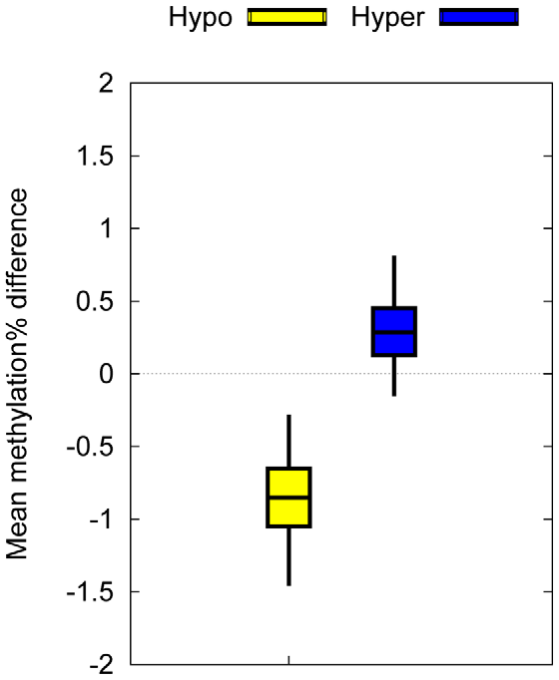
Biological confirmation of the T1D–MVPs in an independent set of T1D–discordant MZ pairs. Mean intra-pair methylation differences associated with T1D–MVPs between 4 T1D–discordant MZ pairs not included in the original dataset. Bars indicate 50% bootstrap confidence intervals on the means, and whiskers indicate 95% confidence intervals on the means. We observed a statistically significant DNA methylation difference in the expected direction between hyper and hypoT1D–MVPs (P = 0.0375, Welch's t-test).

Keeping in mind that the T1D–MVPs displayed intra-pair methylation differences of ∼5%, and that pyrosequencing is limited to identifying inter-sample methylation differences of >5% [22], and not amenable to simultaneously analyzing a large number of different genomic regions, we reasoned that a genome-scale approach would provide greater power for confirming the original T1D–MVPs. Furthermore, confirmation in an independent set of samples would be strong support for the T1D–MVPs being bona fide T1D–assocated epigenetic perturbations. Therefore, as independent biological confirmation of the T1D–MVPs, we performed Illumina27K profiling on CD14^+^ cells obtained from 4 additional T1D–discordant MZ pairs that were not included in the original Illumina27K screen of 15 T1D–discordant MZ pairs. Although this number of MZ pairs is too small to perform de novo T1D–MVP identification, it can be used to test whether the T1D–MVPs found in the first screen show intra-MZ pair DNA methylation differences in the expected direction. Indeed, we observed a statistically significant DNA methylation difference in the expected direction between hyper and hypoT1D–MVPs in these 4 additional pairs (P = 0.0375, Welch's t-test, Figure 3), thus providing biological confirmation of the original T1D–MVP calls.

The successful confirmation of the T1D–MVPs by Illumina27K chips in an independent set of T1D–discordant MZ twins (i.e. genetically identical co-twins) is unlikely to be due to a platform-specific bias. The co-twins for each twin pair were recruited and sampled at the same time, their blood processed simultaneously, and all samples arrayed randomly on the Illumina27K chips. In our opinion, the more likely conclusion is that the T1D–MVP signature is real but of small magnitude, and hence standard bisulfite PCR sequencing-based methods routinely applied for validating methylation differences of relatively larger magnitudes may not be appropriate. Although currently not in routine use, targeted deep coverage high throughput sequencing-based bisulfite sequencing may be a better option for technical validation in future such studies.

### T1D–MVPs are associated with several genes involved in immune function

Each of the 132 different T1D–MVPs is associated with a different gene. Although this number of genes is too small for performing a robust Gene Ontology or pathway analysis, we note that T1D–MVP associated genes or gene products include several known to be associated with T1D or immune responses (Table S2). This includes, the HLA class II gene, *HLA-DQB1*, which carries the highest single genetic risk for T1D (along with *HLA-DRB1*) [23], *RFXAP*, an HLA class II regulating element, *NFKB1A*, an important regulator of apoptosis and inflammatory immune responses, *TNF*, a key inflammatory cytokine associated with T1D in animal models, and *GAD2* which encodes GAD65, a major T1D autoantigen involved in disease etiology [24]. Identification of *GAD2* indicates that we have identified T1D–associated MVPs, and not just an epigenetic signature associated with a non-specific immune response. Also, the T1D–MVPs do not overlap those reported recently for systemic lupus erythematosis in 5 disease-discordant MZ twin pairs [10].

### T1D–MVPs are found in singletons that harbor T1D–associated autoantibodies before disease diagnosis

**Figure 4:**
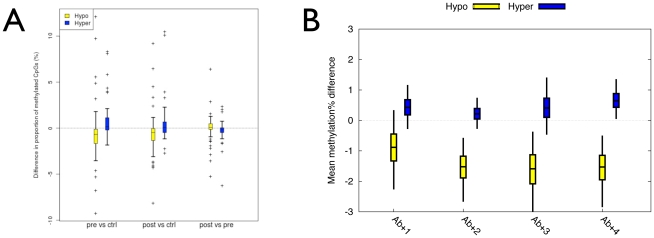
Establishment of the temporal origins and additional independent biological confirmation of the T1D–MVPs. (A) Boxplots of the mean difference in the proportion of CpG sites methylated (%) between 7 pre- or post–T1D diagnosis samples and 18 controls from 9 unaffected MZ pairs or between pre- and post–T1D diagnosis samples at 74 hypo- and 58 hyper-methylated MVPs. Bars indicate 50% bootstrap confidence intervals on the means, and whiskers indicate 95% confidence intervals on the means. (B) Boxplots of the mean difference in the proportion of CpG sites methylated (%) between each of 4 Ab+/T1D–singletons and the same controls as in ‘A’. Bars indicate 50% bootstrap confidence intervals on the means, and whiskers indicate 95% confidence intervals on the means.

The above data do not distinguish among MVPs present before overt T1D, and those caused by insulin treatment or by the disease process after clinical diagnosis. Without making this distinction, it would not be possible to consider T1D–MVPs as potentially causative of the disease. The identification of the temporal origins of disease-associated epigenetic variation thus represents an issue of fundamental importance, but has never previously been addressed in any complex disease epigenomic study [e.g. Refs. 9]–[15]]. To address this issue in the context of T1D, we generated Illumina27K-based DNA methylation profiles for CD14^+^ cells obtained from 7 singletons before and immediately after they presented with clinical T1D. These 7 individuals were recruited from a cohort of healthy school children and young adults of European origin in Alb-Donau County, Germany, all without a family history of T1D [25]. All ‘pre–T1D’ samples were obtained when these individuals had diabetes-associated autoantibodies (GAD65, IA2 and Islet Cell Antibodies), but with normal blood glucose levels and without insulin treatment. We used 9 normal MZ twins pairs as independent controls, since they had not been used for the initial MVP calling. We found that in both pre– and post–T1D samples relative to controls, the same T1D–MVPs display methylation differences in the expected direction (P = 0.015) (Figure 4A and Table S4). Specifically, 71% and 66% of T1D–MVPs showed the expected directionality in pre- vs. control and post- vs. control comparisons respectively. There was no significant difference in T1D–MVP methylation levels between the pre- and post–T1D samples (P>0.6). These results: (i) provide further independent biological confirmation of the original T1D–MVPs (in addition to the 4 different T1D–discordant MZ pairs described above); (ii) demonstrate that T1D–MVPs precede clinical diagnosis; (iii) and show that T1D–MVPs can be identified in the context of normoglycaemia, independent of the disease process, metabolic dysfunction, pharmacological or insulin treatment, or the twinning event since the replication was performed in singletons. It is also important to note that since T1D–MVPs are found both before, and following, T1D–onset within the same individuals, but with no significant difference between pre- vs. post- T1D–onset profiles, we can conclude that at least some T1D–MVPs are temporally stable over many years.

The results described above do not rule out the possibility that T1D–MVPs arise as a result of the sub-clinical autoimmune process that leads to clinical T1D. We therefore performed Illumina27K analysis of 4 different singletons that, at the time of sampling, were autoantibody positive but had still not developed T1D even after 12 years follow up (i.e. Ab+/T1D–, Materials and Methods). Although autoantibody positivity can predict progression to T1D (about 50% progression by 5 years), the longer an individual stays disease-free the lower that risk. The risk of progression to T1D after 12 years from detection of autoantibody positivity estimated at <5% [26]. In a comparison with the same controls used in the analysis of the pre-/post–T1D samples (described above), we found T1D–MVPs to be enriched in the Ab+/T1D–samples (P = 0.0023, Welch t-test, Figure 4B). Specifically, 67% of T1D–MVPs in the Ab+/T1D–singletons, relative to controls, showed directionality consistent with the original calls. This analysis limits the temporal origins of T1D–MVPs to two possibilities. First, they could arise as a result of the autoimmune process associated with the appearance of autoantibodies in the pre–T1D phase, though this process would not be required for persistence of T1D–MVPs, as autoantibodies are infrequent in long-standing T1D (in our study, only 2/15 diabetic co-twins were autoantibody positive at time of sampling). Alternatively, the appearance of T1D–MVPs and autoantibodies could reflect distinct processes. In either case, T1D–MVPs must arise very early in the course of events that lead to clinical T1D.

## Discussion

Disentangling epigenetic effects from the confounding influences of genetic and/or environmental heterogeneity represents a significant barrier to elucidating the etiological role of epigenetic variation in human complex disease. Herein lies the key advance made by our study, as the T1D–MVPs we report here represent the first example of disease-associated epigenetic variation that antedates clinical disease and cannot be explained by genetic heterogeneity, pharmacological treatment, or post-disease cellular dysfunction. Our results provide a platform from which to address several key issues in future studies that we discuss below.

First is the issue of causality. In GWASs, any disease-associated genetic variant is, or linked to, a causative variant. In EWASs, on the other hand, the direction of the cause-consequence relationship is difficult to define if an appropriate study design is not employed. Specifically, the commonly used unrelated singleton “case versus control” design of GWAS is not appropriate as epigenetic variation found to be associated with the disease could simply be due to the disease process itself or disease-associated genetic variation. It is for this reason that we employed the study design described here: T1D–discordant MZ twin pairs combined with longitudinally sampled pre–T1D singletons to rule out genetic differences and establish the temporal origins of T1D–associated epigenetic variation. Using this approach, we were able to demonstrate that T1D–MVPs antedate clinical disease. However, it will be important to further explore the temporal origins of the T1D–MVPs by analyzing samples obtained before the appearance of T1D–associated autoantibodies, which could help determine whether T1D–MVPs arise even before the sub-clinical immune phase. In this regard, longitudinal birth cohorts will be invaluable [27]. If some T1D–MVPs were found before the sub-clinical immune autoantibody response, then the hypothesis that these T1D–MVPs are causing disease would be strengthened. The identification of T1D–MVPs in individuals before the appearance of T1D–associated autoantibodies would exclude T1D–MVPs being simply secondary to the autoantibody-associated immune process.

Establishing the temporal origins of T1D–MVPs will also be useful for elucidating the biological origins of T1D–MVPs. For example, if there were evidence that T1D–MVPs exist at birth (e.g. from birth-cohort studies), then this would suggest stochastic or environmental factors that operate *in utero*. Given that we have studied MZ twins—genetically identical individuals exposed to similar environments during childhood—early life stochastic origins of T1D–MVPs is an attractive idea. Indeed, stochastic epigenetic variation in humans is more common than previously appreciated as demonstrated by the recent genome-scale analysis of DNA methylation profiles in 114 monozygotic (MZ) and 80 dizygotic (DZ) twins [11]. A potential source of stochastic epigenetic variation could be genetic variants that increase the probability of stochastic epigenetic variation *in cis*, as suggested by various authors [28]–[30]. In the context of our results, it doesn't mean that T1D–MVPs are due to somatic genetic differences, but rather the T1D–discordant twins may harbor germline genetic variants that are associated with increased levels of epigenetic stochasticity, and indeed we find that T1D–MVPs are less epigenetically variable in the normal MZ twins (Figure 2). If this occurs in the context of a genomic background that is predisposed to a given disease, then it could impact on the probability of one twin developing the disease, whereas the co-twin remains disease-free. However, it is also possible that T1D–MVPs are induced environmentally as MZ twins are exposed to similar, but not identical, environments and there are examples of disease-relevant environmental factors that operate in early life to influence disease-risk [31]. Given a large enough sample size and genome-coverage, it might be possible to identify environmental triggers based on gene regulatory networks enriched for T1D–associated epigenetic and transcriptional variation.

Third, although we have focused on promoter-associated single CpGs here, our data suggest that larger surrounding genomic regions are affected (i.e. differentially methylated regions or DMRs), and it will be important to further define these regions spatially. In the near future, it should be possible to perform high throughput sequencing-based whole-genome DNA methylomic profiling in large cohorts to: (i) identify new T1D–MVPs/DMRs, including those that might exist outside of promoter regions; (ii) help define the boundaries of the T1D–associated DMRs, if they exist; (iii) establish the hierarchy of CpG sites within a DMR in terms of functional impact, that is, it is possible we have identified T1D–MVPs that are ‘linked’ to the most discriminative CpG site i.e. a ‘tag’-MVP, similar to tag-SNPs in GWASs; (iv) profile a number of key cell types including other immune effector cells.

Fourth, we need to understand the functional outcome of T1D–MVPs at the molecular level. The most obvious impact is on gene expression, but equally important will be investigations into how the MVPs alter the local chromatin structure. For example, do they alter the binding of key transcription factors? Or do they correlate with alterations in other epigenetic marks such as histone modifications? The magnitude of methylation differences we have identified at T1D–MVPs is relatively small compared with DNA methylation perturbations generally observed in the context of cancer. However, given that other small-scale studies of non-malignant disease-associated methylation variation in humans also report effects of small magnitude [10]–[15], it is quite possible that this is the norm for complex disease-associated epigenomic variation. In this regard, it is worth drawing parallels with findings from GWAS in which most variants individually confer a small disease-risk [32]. Therefore, studying the local chromatin architecture and gene expression will help define how DNA methylation variants of small magnitude impact on molecular outcomes in a variety of key immune effector cells, thus helping to elucidate how T1D–MVPs, in combination with genetic and other environmental factors, are involved in T1D etiology and the causal or consequential nature of the T1D–MVPs. Of course, it is also quite possible that some T1D–MVPs are not directly involved in the T1D pathogenesis process, but rather are biomarkers for the disease. This is similar to T1D–associated GAD65, IA2 and islet cell autoantibodies, which are highly predictive of disease, but without evidence that they are involved in T1D etiology. Analysis of individuals before they present with autoantibodies will be key to establishing whether T1D–MVPs are valuable biomarkers for the disease that can augment the predictive power of autoantibodies and genetic variants.

It is noteworthy that a T1D–MVP signature was detected by assaying a relatively modest number of samples and genome coverage, which emphasizes the power of our study design that combines MZ twins and prospectively sampled individuals, as opposed to the typical singleton ‘case versus control’ approach. Although previous complex disease epigenomic studies have correlated disease-associated epigenetic variants with changes in gene expression or temporal stability [10]–[15], none have been able to address the key question of temporal origins, which is critical for establishing the direction of the cause-consequence relationship between disease phenotype and epigenetic variation. Therefore, in addition to identifying a previously unappreciated molecular component of type 1 diabetes risk, we believe our study also represents one possible blueprint for future EWASs of other complex diseases [33].

## Materials and Methods

### Processing of monozygotic twin samples

Monozygotic twin pairs were selected from the British Diabetic Twin Study (Table S1) [34]. Twins are ascertained by referral through their physicians. Twins were genotyped for *HLA-DQB1* and -*DRB1* using a sequence-specific oligonucleotide probe–based method and line strips from Roche Molecular Systems. Diabetes-associated autoantibodies were analyzed as described below. Monozygosity was established using both clinical data and DNA fingerprinting (data not shown). T1D was defined according to the National Diabetes Data Group criteria and diabetes excluded in the non-diabetic co-twins by a 75 g oral glucose tolerance test and random whole blood glucose at testing less than 7.0 mmol/L [34]. All T1D patients were treated from diagnosis with insulin and take highly purified human insulin at least twice daily. All subjects gave informed consent and the study was approved by the Northern and Yorkshire Research Ethics committee (REC Reference Number: 06-MREO-3-22). PBMCs from subjects investigated were prepared from heparinised blood using standard Ficoll-Hypaque separation. After washing, PBMCs were counted using the Naebaur counting chamber after staining with Trypan blue to asses cell viability, and washed in PBS before isolation of CD14^+^ cells. Briefly PBMCs were washed with 10 ml MACS buffer and CD14^+^ cells isolated according to manufacturer's instruction using positive selection MS column (Miltenyi Biotec, UK, # 130-042-201). From each MACS-enriched CD14^+^ cell sample, we took two aliquots (1×10^5^ cells in each case) and stained with either mouse IgG as a negative control or CD14^+^ FITC. CD14^+^ percentage purity was determined by FACS (Figure S1). Overall percentage purity of CD14^+^ cells observed typically ranged from 90–95%. CD14^+^ cells were then lysed for DNA extraction using Qiagen method according to manufacturer's instructions. The concentration of DNA was determined by using NanoDrop.

### Processing of pre–T1D, post–T1D, and Ab+/T1D–singletons

These singletons were recruited from a cohort of healthy school children and young adults of European origin in Alb-Donau County, Germany [31]. All subjects screened were without a family history of T1D. Of 7287 children, 72 children had islet cell autoantibodies and were followed prospectively. Of these 72, 10 developed T1D, of whom 7 had relevant blood samples available for analysis both initially and at diagnosis (Table S1). Samples were analyzed for HLA-A, HLA-B, HLA-DRB1 and HLA-DQB1 genotypes and diabetes-associated autoantibodies as for the MZ twins. Blood was obtained from 7 subjects at two time points, before and after T1D diagnosis, for replication of T1D–MVPs. PBMCs were isolated from whole blood by standard Ficoll–Hypaque density gradient centrifugation. Briefly, approximately 10 mL of heparinized, plasma-reduced blood was diluted with Hank's buffered salt solution (HBSS; 1∶2 dilution). Then, 15 mL of Ficoll was covered with a layer of diluted blood (30 mL). After 30 min of centrifugation (2000 rpm, room temperature), the PBMCs could be easily collected. After two washing steps and cell counting, the isolated PBMCs were frozen and stored in a liquid nitrogen freezer. PBMCs were frozen in FBS containing 10% dimethylsulfoxide (DMSO). Several portions of a given blood sample were frozen, typically a cryotube vial contained between 8–12 million PBMC each. For the isolation of CD4^+^, CD8^+^ and CD14^+^ cells, PBMCs were thawed and washed in 10 mL of prewarmed PBS to remove all traces of the cryoprotectant in the freezing medium. Cell viability was determined spectrophotometrically using trypan blue staining. After thawing, cells were obtained and stained with trypan blue solution 0.4%. Finally, at least 200 cells were counted under the microscope. For magnetic separation only cryo-preserved samples were used. Since Magnetic beads may bind to dead cells non-specifically, only samples that show a cell viability of more than 90% were analyzed. Using this criterion of initially 20 PBMC samples 15% had to be excluded. Cell viability as determined by trypan blue exclusion was not dependent on storage time in liquid nitrogen. Magnetic separation was performed according to the manufacturers specifications (MACS, Miltenyi Biotec). To evaluate the efficiency and purity (typically ≥95%) of the magnetic separation flow cytometry analysis was performed after cell separation using standard staining protocol for surface markers. All subjects gave informed consent and the study was approved by the local ethical committee (ref: 08/1990 & 07/1998).

### T1D–associated autoantibodies

All twin samples were tested at a single laboratory (London) in batched assays as previously described [33]. Positive results were duplicated to limit the false positive rate to less than 0.2%. In the latest 2010 Diabetes Antibody Standardization Program (DASP) the London assay characteristics were: GADA sensitivity 82%, specificity 86%; I-A2A, sensitivity 60%, specificity 98%; and ZnT8A sensitivity 72%, specificity 88% (data unpublished). Islet Cell autoantibodies was performed in Ulm, Germany in a batched assay as described (30) and samples were also tested in London, UK for GADA, I-A2A and ZnT8A [35]. Islet Cell autoantibodies were measured by indirect immunofluorescence, with detection limit 5 JDF units, and >20 JDF units as positive; assay sensitivity and specificity was 100% in 13th Islet Cell Autoantibody Workshop (1998); results were documented as positive or negative.

### Array processing

Arrays were processed at the Barts and The London Genome Centre, London, UK according to the manufacturer's recommendations. Methylation scores for each CpG site are called as ‘Beta’ values (using BeadStudio software from Illumina), that range from 0 (unmethylated, *U*) to 1 (fully methylated, *M*) on a continuous scale, and are calculated from the intensity of the *M* and *U* alleles as the ratio of fluorescent signals b = *Max*(*M*,0)/(*Max(M*,0)+*Max(U*,0+100). For many arrays, a small number of probes do not yield sufficient signal for BeadStudio to make a Beta value score. Samples where more than 5% of probe Beta values were missing were discarded or repeated. We also checked the distribution of Beta values for the expected bimodal distribution of Beta values, and repeated arrays with <10% of probe Beta values > = 0.75. We also discarded any probes for which a score was missing for any array in the final set of arrays, and probes located on the X- and Y-chromosomes. The final dataset therefore comprised of 22,645 (of the total 27,458) CpG sites. The raw Beta scores were normalized using standard quantile normalization algorithms that are available from https://github.com/dasmoth/metharray-scripts.

### Identification of T1D–MVPs

For each of the 22,645 CpG probes for which we had complete data, normalized Beta scores from 15 diabetics were compared with those from their normal twins using the Wilcoxon signed-rank test. Given the sample size and number of probes under consideration, it was not possible to reasonably correct for multiple testing, so we adopted a pragmatic approach, using the uncorrected p-values from this test as an indicator of possible T1D–MVPs which were then validated by subsequent analyses. All probes with a Wilcoxon p-value<0.01 were considered to be potential T1D–MVPs.

### Pyrosequencing validation

One µg of DNA was bisulfite converted using the EpiTect-96 Bisulfite kit (Qiagen, Hilden, Germany) according to the manufacturer's instructions. Regions of interest for validation were amplified using 30 ng of bisulfite treated human genomic DNA and 5 to 7.5 pmol of forward and reverse primer, one of them being biotinylated. Sequences for oligonucleotides for PCR amplification and pyrosequencing are listed in Table S5. Reaction conditions were 1× HotStar Taq buffer supplemented with 1.6 mM MgCl_2_, 100 µM dNTPs, and 2.0 U HotStar Taq polymerase (Qiagen) in a 25 µl volume. The PCR consisted of a denaturing step of 15 min at 95°C followed by 50 cycles of 30 s at 95°C, 30 s at the respective annealing temperature and 20 s at 72°C, with a final extension of 5 min at 72°C. 10 µl of PCR product were rendered single-stranded and 4 pmol of the respective sequencing primer were used for analysis. Quantitative DNA methylation analysis was carried out on a PSQ 96MD system with the PyroGold SQA Reagent Kit (Pyrosequencing) and results were analyzed using the Q-CpG software (V.1.0.9, Pyrosequencing AB). From the original 30 different pyrosequencing amplicons, we discarded those that did not have complete data for all 16 pairs. For the 17 remaining amplicons (7 hyper-MVPs, 10 hypo-MVPs) we calculated the mean difference between all CpGs in the amplicon for all T1D vs. all normal samples in the set.

### Analysis of T1D–MVPs in independent MZ pairs and pre/post–T1D and Ab+/T1D–singletons

Mean methylation differences associated with each T1D–MVP for which complete data was available (Table S2) were calculated between 4 T1D–discordant MZ pairs not included in the original dataset, or between 4 Ab+/T1D–singletons and 9 singletons representing the control pair. This yields a single overall methylation difference for each T1D–MVP. Significance of differences between hyper- and hypo-MVPs was determined by Welch's t-tests between scores associated with hyper-MVPs and scores associated with hypo-MVPs.

For the pre/post–T1D samples, we compared methylation in pre/post–T1D samples and controls using one-sided Wilcoxon rank sum tests, with the alternative hypothesis defined so that direction of methylation difference between pre/post and control samples was the same as between affected and unaffected MZ pairs. The expected within MZ pair correlation causes the variance to differ in the two groups when comparing pre/post samples to controls, which is not allowed for using a rank sum test. An alternative is to use a non-parametric test tailored to this Behren's-Fisher problem, and we found P values were similar between the two tests where tests could be performed, but that the rank sum tended to be conservative at smaller p values. However, the nonparametric Behrens-Fisher statistic is not computable for the most extreme scenarios, when all values in one group exceed all values in another. Therefore we chose to use the rank sum test in the knowledge results would be conservative. We used signed rank tests to compare differences in pre and post–T1D samples. In both cases, we summarized the evidence that there was differential methylation using X = mean(−log(p)) with the mean taken over the T1D–MVP sites. Under the null hypothesis, −log(p) should have an exponential distribution with mean 1, and X may therefore be interpreted as the scale parameter describing this exponential distribution, and values above 1 indicate smaller p values than would be expected under the null. To avoid assuming independence between T1D–MVP sites (an assumption which can be problematic with array based assays), we estimated bootstrap confidence intervals by re-sampling seven pre/post samples and nine control MZ pairs with replacement 100,000 times.

## Supporting Information

Figure S1 Comparison of Illumina27K profiles with bisulfite PCR sequencing data from the Human Epigenome Project (HEP). For every probe on the Illumina methylation array lying within 100 bp of an assayed region in the HEP bisulfite dataset, we compared the mean Illumina Beta score across the control CD4 samples to the mean HEP methylation level averaged across all CpG sites lying within 100 bp of the Illumina probe. The correlation between the two datasets is R^2^ = 0.88 (Pearson's).
(DOC)

Figure S2 FACS analysis for CD14^+^ purity following Magnetic Activated Cell Sorting (MACS) enrichment. From each MACS-enriched CD14^+^ monocyte cell sample, we took two aliquots (1×105 cells in each case) and stained with either mouse IgG as a negative control (A) or CD14^+^ FITC (C). CD14^+^ percentage purity was then determined by FACS analysis on gated P2 as shown in (B) for the mouse IgG aliquot, and (D) for the CD14^+^ FITC aliquot. Percentage purity for each case is shown in the tables on right for both. Overall percentage purity of CD14^+^ cells observed typically ranged from 90–95% (refer to ‘P2’ %Parent values listed in the table). Shown is a representative example (T1Dpair8-affected).
(DOC)

Figure S3 Pyrosequencing validation of T1D–MVPs. Mean methylation differences between 15 diabetics and their healthy twins in bisulfite-pyrosequencing amplicons around hypo-MVPs (n = 8) and hyper-MVPs (n = 5). Bars indicate 50% bootstrap confidence intervals on the means, and whiskers indicate 95% confidence intervals on the means.
(DOC)

Table S1 Sample information.
(DOC)

Table S2 List of T1D–MVPs called at P<0.01. T1D–MVPs were called in the 15 T1D–discordant MZ pairs using pairwise T-tests as described in the Supplementary Methods (above). The CpG ID is the Illumina27K probe reference for the CpG in question. HypoT1D–MVPs are coloured in yellow and HypoerT1D–MVPs in blue. P-values are derived from pairwise T-tests. The methylation values = 100*(Beta value of affected twin – beta value of unaffected twin).
(DOC)

Table S3 Pyrosequencing analysis of selected T1D–MVPs. Only the 13 different reactions for which we obtained data for all samples are shown. Blue = Hyper-T1D–MVPs and Yellow = Hypo-T1D–MVPs.
(DOC)

Table S4 Independent replication and establishment of the temporal origins of T1D–MVPs. For each T1D–MVP, we used Wilcoxon rank sum tests to compare methylation in seven pre-/post–T1D diagnosis samples to 18 samples from the nine unaffected MZ control pairs (see Methods). Under the null hypothesis, the −log(p) values from this test follow an exponential distribution, with scale parameter 1; values above 1 indicate that p values tend to be smaller than expected under the null. The table shows the estimated scale parameter, bootstrap 95% confidence intervals and one-sided P values calculated by inverting the one sided bootstrap confidence interval with leftmost limit equal to one (i.e. with an alternative hypothesis defined by smaller p values than expected under the null) derived from 100,000 bootstraps.
(DOC)

Table S5 List of primers used in the pyrosequencing assays.
(DOC)
