# Supplementary figures and images for: Identification of Type 1 Diabetes–Associated DNA Methylation Variable Positions That Precede Disease Diagnosis
Source: PLoS Genet. 2011 Sep 29;7(9):e1002300. doi: 10.1371/journal.pgen.1002300 (PMC3183089; doi:10.1371/journal.pgen.1002300)

**
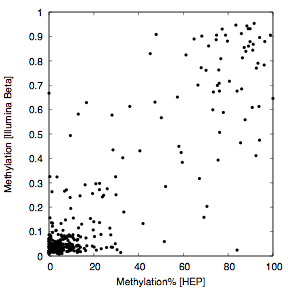
**

Supplement: Figure S1 — Comparison of Illumina27K profiles with bisulfite PCR sequencing data from the Human Epigenome Project (HEP). For every probe on the Illumina methylation array lying within 100 bp of an assayed region in the HEP bisulfite dataset, we compared the mean Illumina Beta score across the control CD4 samples to the mean HEP methylation level averaged across all CpG sites lying within 100 bp of the Illumina probe. The correlation between the two datasets is R2 = 0.88 (Pearson's). (DOC) [file pgen.1002300.s001.doc]

**
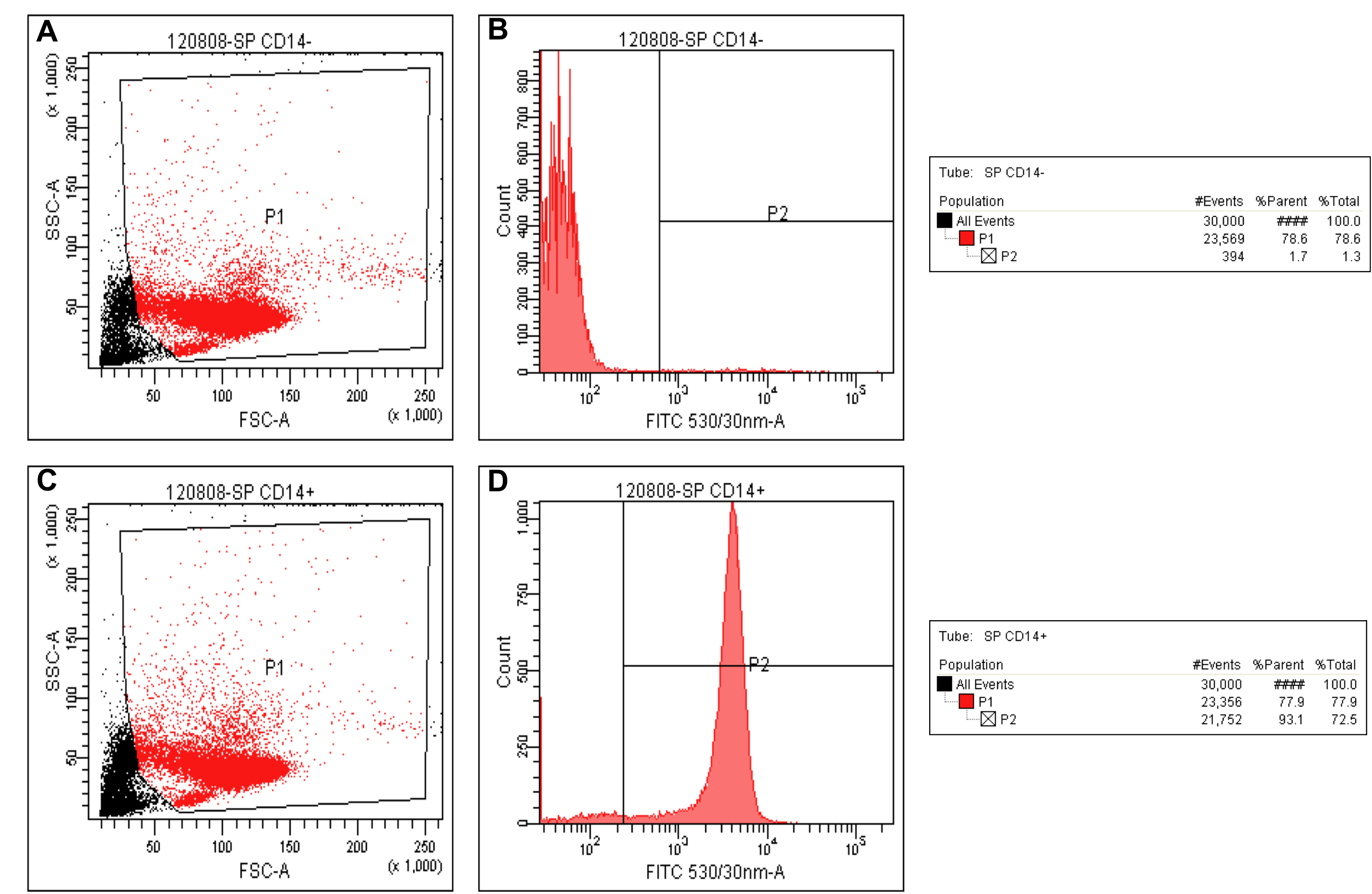
**

Supplement: Figure S2 — FACS analysis for CD14+ purity following Magnetic Activated Cell Sorting (MACS) enrichment. From each MACS-enriched CD14+ monocyte cell sample, we took two aliquots (1×10^5^ cells in each case) and stained with either mouse IgG as a negative control (A) or CD14+ FITC (C). CD14+ percentage purity was then determined by FACS analysis on gated P2 as shown in (B) for the mouse IgG aliquot, and (D) for the CD14+ FITC aliquot. Percentage purity for each case is shown in the tables on right for both. Overall percentage purity of CD14+ cells observed typically ranged from 90–95% (refer to ‘P2’ %Parent values listed in the table). Shown is a representative example (T1Dpair8-affected). (DOC) [file pgen.1002300.s002.doc]

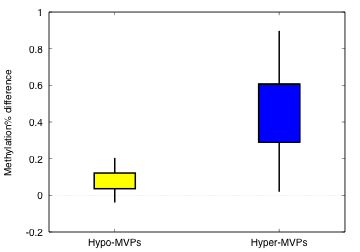

Supplement: Figure S3 — Pyrosequencing validation of T1D–MVPs. Mean methylation differences between 15 diabetics and their healthy twins in bisulfite-pyrosequencing amplicons around hypo-MVPs (n = 8) and hyper-MVPs (n = 5). Bars indicate 50% bootstrap confidence intervals on the means, and whiskers indicate 95% confidence intervals on the means. (DOC) [file pgen.1002300.s003.doc]

**
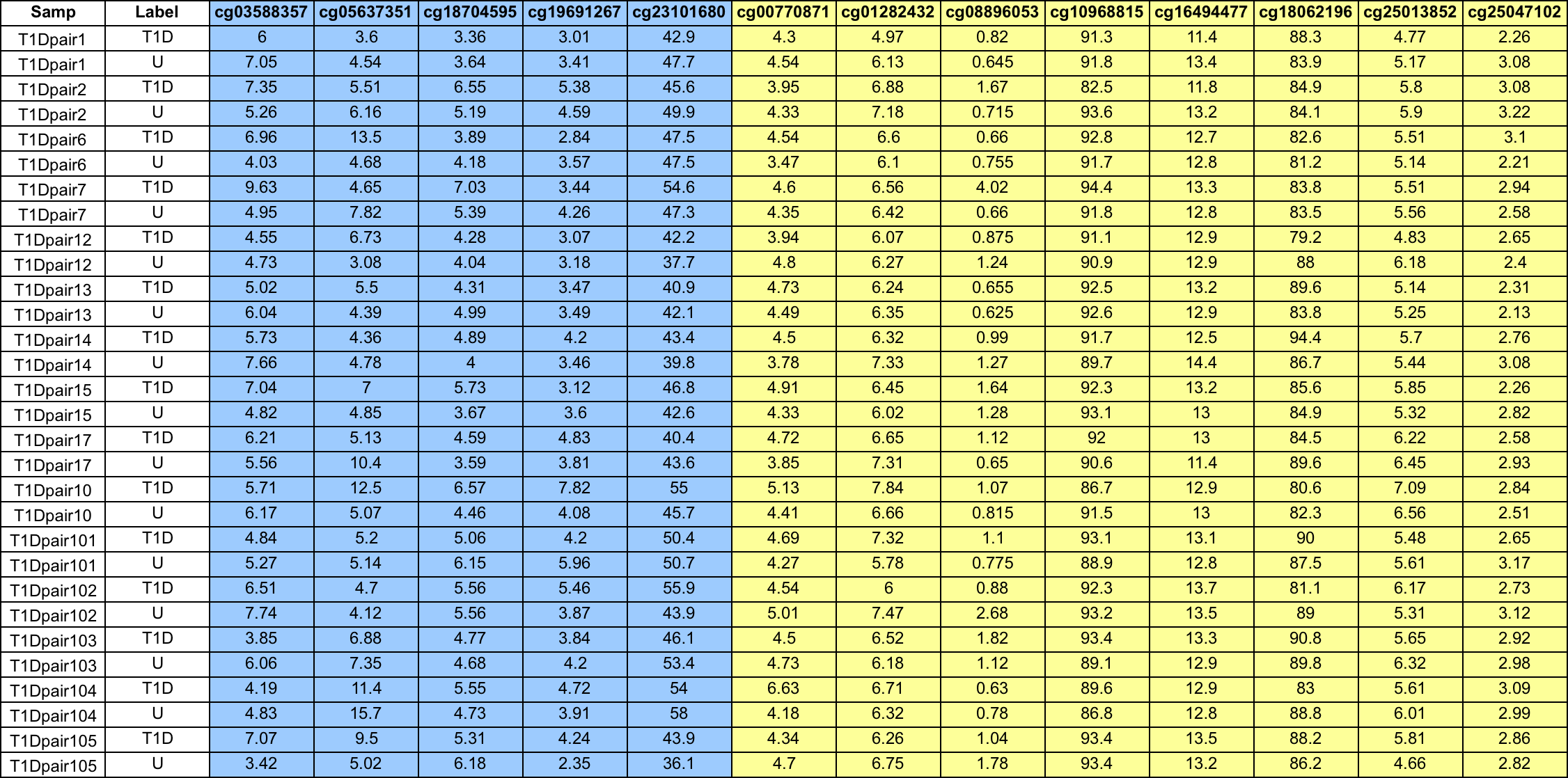
**

Supplement: Table S3 — Pyrosequencing analysis of selected T1D–MVPs. Only the 13 different reactions for which we obtained data for all samples are shown. Blue = Hyper-T1D–MVPs and Yellow = Hypo-T1D–MVPs. (DOC) [file pgen.1002300.s006.doc]

**
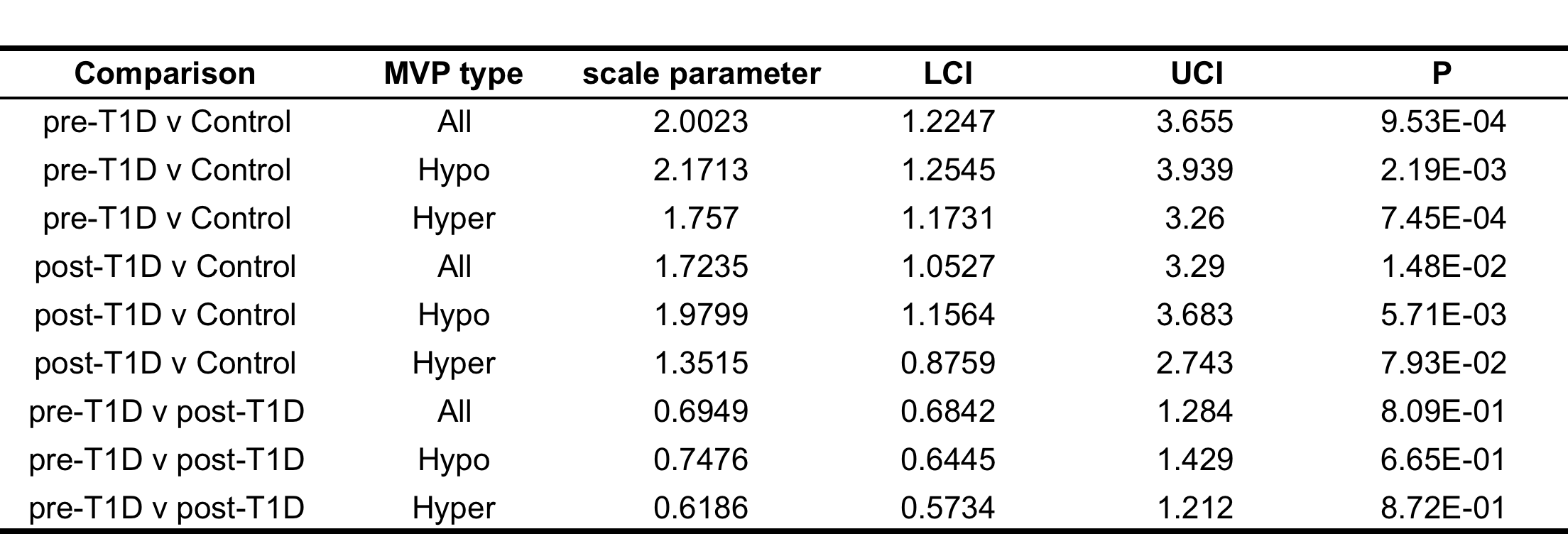
**

Supplement: Table S4 — Independent replication and establishment of the temporal origins of T1D–MVPs. For each T1D–MVP, we used Wilcoxon rank sum tests to compare methylation in seven pre-/post–T1D diagnosis samples to 18 samples from the nine unaffected MZ control pairs (see Methods). Under the null hypothesis, the −log(p) values from this test follow an exponential distribution, with scale parameter 1; values above 1 indicate that p values tend to be smaller than expected under the null. The table shows the estimated scale parameter, bootstrap 95% confidence intervals and one-sided P values calculated by inverting the one sided bootstrap confidence interval with leftmost limit equal to one (i.e. with an alternative hypothesis defined by smaller p values than expected under the null) derived from 100,000 bootstraps. (DOC) [file pgen.1002300.s007.doc]
