# Supplementary material for: Identification of Type 1 Diabetes–Associated DNA Methylation Variable Positions That Precede Disease Diagnosis
Source: PLoS Genet. 2011 Sep 29;7(9):e1002300. doi: 10.1371/journal.pgen.1002300 (PMC3183089; doi:10.1371/journal.pgen.1002300)
Supplement: Table S5 — List of primers used in the pyrosequencing assays. (DOC) [file pgen.1002300.s008.doc]

| **CG** | **Size** | **PCR primer forward** | **PCR primer reverse** | **Pyrosequencing primer** |
| --- | --- | --- | --- | --- |
| **cg19046959**  chr1:36,565,657-36,566,105 | 218 | Biotin-TATTTAGGGTTGGTTTGATGATTTT | TCCCACAAATCCTTTCTACATAAAC | TCAAAATCCCCAACAT |
| **cg11480873**  chr1:205,744,217-205,744,665 | 255 | Biotin-AAAAAGGAAATTTTGTATTTAATTTATA | AAAAAAAATCCCCATCATCTAAC | TCAATCACTAACTTAAAACC |
| **cg08466883**  chr1:215,741,118-215,741,566 | 163 | GAGGTTAAGTTTGGAGGGGAA | Biotin-CATTCTAAAACAACAACTACTCTACCAC | GGAAGGGAGAGGTAGAAG |
| **cg13982661** chr11:121,163,250-121,163,698 | 231 | Biotin-GTTTTTATTTGGAGGTGGTT | CCCCTTAATCAAAAAACAAAAAAA | ACAATTTACAAAACAATAAC |
| **cg23101680**  chr13:46,277,038-46,277,486 | 197 | GGTGTGTTGGGGAAGGTTATATAG | Biotin-CCCAAAAATCCAAAATAATTACAC | GTTTTTATGATATATTGAAG |
| **cg19691267** chr13:41,558,288-41,558,318 | 191 | GTTTTTGATTGGTTAGTTGGAGGT | Biotin-CTAAAAAACACACCCTAAAAACCC | GGGAAGATAAGGAGGG |
| **cg03588357** chr14:91,719,925-91,720,373 | 152 | Biotin-GGTGGTTTGGGGAGGTTTT | CCAAACCTAAAAACTATAATCAAC | AAACACAACTTAACCCTAC |
| **cg21413009** chr15:64,338,748-64,339,196 | 222 | TGGTGGGGAGAGGAGTATATAAGTAT | Biotin-AAACACCTCAAAACTCCCAAAC | TTTTGAGGATTGTGTTAGA |
| **cg13843603** chr17:30,771,794-30,772,242 | 218 | Biotin-TGGGAGGGTTTGAGGTAATTT | CCAAAACCTTTCAAATAAAAACTCTAA | TTCAAATAAAAACTCTAAAA |
| **cg05637351** chr18:63,417,422-63,417,870 | 172 | GTGAAAGTAGGAGGGGTTGTAGTT | Biotin-AACTTTACCTTCATACCTCAAAAACTC | TAATATTTATTTTAGTTTGT |
| **cg20673481** chr2:18,059,206-18,059,654 | 159 | Biotin-GGAATAAGATAAAGTATTTTGTTTTGTATT | TTTACCTAAATTTCCTCAAAAAATC | CTAAATTTCCTCAAAAAATC |
| **cg17607231** chr2:231,090,082-231,090,530 | 148 | AAAAGTGAAATTGTTTAATTAGTTGA | Biotin-TAACAAAAACAAATAACCCTATCTT | GAGGAGGAGTAGAGTTAGTT |
| **cg18704595** chr20:43,280,758-43,281,206 | 208 | GTTGTTGTTTGGTTGAAGTTTATTT | Biotin-CCTCCTAAACATTCACCTTTAAAAA | ATTTTGTATAGGTTTTTGG |
| **cg25791888** chr21:45,079,832-45,080,280 | 300 | Biotin-AGTTGAGAAAAGATTTATATGGGTT | TTCCCAAAATACTAAAATTACATAC | TCTATCTTCTCTCCTACAAC |
| **cg14013300**  chr22:29,195,847-29,196,295 | 243 | Biotin-TAGTAAGAGGGTTTGGAAGATAGGT | AAAAACAAAAACAAAAACTAAAAAC | TTTTCTATACTTTTTAACTC |
| **cg27437944** chr3:180,630,286-180,630,734 | 228 | Biotin-GAGTTTTGGTTTGGTTGTTTTTT | CTCCTCCCCTCTAATTAAAAAATTT | CTTTCTACAAAATACTAACC |
| **CG** | **Size** | **PCR primer forward** | **PCR primer reverse** | **Pyrosequencing primer** |
| **cg10705800**  chr1:41,328,221-41,328,669 | 196 | GGTTATGGGAATGGATAGTTGATT | Biotin-CCCCAAAAACTACAAAATATCAAAC | GTGTATTTTTAGGAAGTTTT |
| **cg04599297**  chr10:26,505,243-26,505,691 | 189 | Biotin-GGGATTATTTAAGGGTTTT | CCCCTCACTCTAACTCCAACTAAC | CTCAAATACTCTAAAACTCT |
| **cg19596755** chr11:68,609,547-68,609,995 | 275 | ATGTTTTAGGTATTTTTTGATGGTATTTAT | Biotin-AAAATCAAATCTAAACCCAATATCC | TTTTTATGTAGATAAGGGT |
| **cg00770871**  chr12:2,999,641-3,000,089 | 274 | GTTAGAGTGGTTAGGTTGGTA | Biotin-AAACCCAAACAAAAACAAAATCTT | GGTTTGGTTTTTTGGA |
| **cg06255524** chr13:96,328,689-96,329,137 | 200 | GGGATTATTTGATTTTTTTTAGGTT | Biotin-ACTCAATTCAAACAATATTTATTCC | TTGGAATTGGGGTTT |
| **cg01282432**  chr14:57,046,658-57,047,106 | 146 | GTGGGTTTTGGAATATATAATAGTG | Biotin-AAAATTACCCCTTCTATCCCTCT | AGGTTTAGAGTTGATTAGAT |
| **cg25047102** chr16:31,106,118-31,106,566 | 223 | GTTTTTTTTGGTTTTTTGATT | Biotin-CTTCATTTCCCTTAATACCTTACAAC | GGTATGATGGGAGTTGTAGT |
| **cg18062196** chr17:57,642,427-57,642,875 | 205 | GGGAAATGAATAGTTTTGGTGATT | Biotin-AACTATTCTACCTTCACACACTTAAC | TTGGGATTATGGTTTTAGT |
| **cg22271212**  chr19:3,028,539-3,028,987 | 211 | AGGATGTATTTTTAGGGAAGGTATT | Biotin-CCCCTCACCTATAATATTAAACCTA | GGAATTTAGATTTTGA |
| **cg10968815** chr20:31,595,098-31,595,546 | 103 | TGTTTAGTTTTAGGGGTTTGTAGTG | Biotin-ATAACCTTTACCAAAATATCTCATCC | TGGGTTTGGTTATTGG |
| **cg03835292** chr22:37,976,431-37,976,879 | 200 | TTATTTTGGTTTTTTAAAGTGTTGG | Biotin-ATAAATAATTTAAAATCTTTTCTTTAAATC | AAAGTGTTGGGATTATAGG |
| **cg16494477** chr5:170,847,052-170,847,500 | 225 | Biotin-GATAGTTAGGTAAGGTGGGTAGTTT | CAACAAAATCTACAACAACAACAAC | CCTACACTTACCTATAA |
| **cg18075454** chr6:153,323,975-153,324,423 | 205 | Biotin-TGGATAAGAGAAATATAAGTAGGAAAAA | AAATTTCTTTCCCATACACTAAATC | AAACCTCCAAAACC |
| **cg08896053**  chr8:50,823,269-50,823,717 | 202 | Biotin-AGGTAGTTTGGAGGTTTAGGGTAGT | AAATAAACTAAAAAACCAACAAAAA | TTCTACCCCCACTACAA |
