# Supplementary material for: Identification of Type 1 Diabetes–Associated DNA Methylation Variable Positions That Precede Disease Diagnosis
Source: PLoS Genet. 2011 Sep 29;7(9):e1002300. doi: 10.1371/journal.pgen.1002300 (PMC3183089; doi:10.1371/journal.pgen.1002300)
Supplement: Supplementary file 3 [file pgen.1002300.s004.doc]

T1D-discordant MZ pairs

Sample ID	Illumina27K array ID	Sex	Age at onset	Disease duration at sampling	Age at sampling	GAD	IA2	ZnT8	HLA A*a	HLA A*b	HLA B*a	HLA B*b	HLA DRB1*a	HLA DRB1*b	HLA DQB1*a	HLA DQB1*b
T1Dpair1 - U	4635311084_F	F			25	Negative	Negative	Negative	0301	2902	4403	5101	0101	0401	0302	0501
T1Dpair1 - A	4635311088_D	F	10	15	25	Negative	Negative	Negative								
T1Dpair2 - U	4635311084_I	F			52	Negative	Negative	Negative	0201/0224	0201/0224	1501	4402	-	-	0301	0302
T1Dpair2 -A	4635311047_D	F	13	39	52	Negative	Negative	Negative								
T1Dpair3 - U	4635311075_B	F			39	Negative	Negative	Negative								
T1Dpair3 - A	block6_a8	F	18	21	39	Negative	Negative	Negative	0101	0201/0224	0801	5101	0301	0408	0201	0301
T1Dpair4 - U	4635311043_H	F			16	Negative	Negative	Negative	2402	2402	3906	5501	0401	0801	0302	0402
T1Dpair4 - A	4635311043_I	F	2	14	16	Negative	Negative	Negative								
T1Dpair6 - U	4635311043_J	M			36	Negative	Negative	Negative	0301	3101	0702	3501	0413	1501/1502	0302	0602
T1Dpair6 - A	4635311043_A	M	13	24	36	Negative	Negative	Negative								
T1Dpair7 - U	4635311088_K	F			42	Negative	Negative	Negative	0201/0224	0201/0224	-	-	0301	0404	0201	0302
T1Dpair7 - A	4635311075_E	F	14	28	42	Negative	Negative	Negative								
T1Dpair8 - U	4635311066_E	F			20	Negative	Negative	Negative	0101	0101	0801	1501	0301	0401	0201	0302
T1Dpair8 - A	4635311084_A	F	7	13	20	Negative	Negative	Negative								
T1Dpair10 - U	4635311066_D	M			33	Negative	Negative	Negative	0201/0224	3002	-	-	-	-	-	-
T1Dpair10 - A	4635311065_K	M	5	27	33	Negative	Negative	Negative								
T1Dpair11 - U	4635311043_C	M			25	Negative	Negative	Negative	ND	ND	ND	ND	ND	ND	ND	ND
T1Dpair11 -A	4635311075_G	M	6		25	Negative	Positive	Negative	ND	ND	ND	ND	ND	ND	ND	ND
T1Dpair12 - U	block6_a10	M			50	Negative	Negative	Negative	0201/0224	0301	1801	4402	0301	0901	0201	0301/0303
T1Dpair12 - A	block6_a3	M	12	34	50	Negative	Negative	Negative								
T1Dpair13 - U	block6_b8	F			43	Negative	Negative	Negative	0101	0201/0224	4001	4402	[0407-0402]	[1301-1302]	-	-
T1Dpair13 - A	block6_b12	F	11	32	43	Negative	Negative	Negative								
T1Dpair14 -U	block6_a11	F			68	Negative	Negative	Negative	0101	0301	0702	0801	0101	0301	0201	0501
T1Dpair14 -A	block6_b1	F	17	51	68	Negative	Negative	Negative								
T1Dpair15 -U	4635311047_H	F			37	Negative	Negative	Negative	0101	2601	1801	3801	0301	0404	0201	0302
T1Dpair15 - A	4635311066_K	F	10	27	37	Negative	Negative	Negative								
T1Dpair16 - U	4635311065_B	M			52	Negative	Negative	Negative	2902	3301	0801	1402	0102	0301	0201	0501
T1Dpair16 - A	4635311066_F	M	20	33	52	Negative	Negative	Negative								
T1Dpair17 - U	4635311065_I	F			42	Negative	Negative	Negative	0201/0224	2402	0702	3906	0801	1302	-	-
T1Dpair17 - A	4635311066_B	F	8	34	42	Positive	Negative	Negative								

Normal MZ pairs

Sample ID	Illumina27K array ID	SEX	Age at sampling	GAD	IA2	ZnT8	HLA A*a	HLA A*b	HLA B*a	HLA B*b	HLA DRB1*a	HLA DRB1*b	HLA DQB1*a	HLA DQB1*b
Normalpair1-1	4635311072_J	F	30	Negative	Negative	Negative	0201	2902	-	-	0701	1501	0202	0602
Normalpair1-2	4635311072_A	F	30	Negative	Negative	Negative								
Normalpair2-1	batch4_d2	F	51	Negative	Negative	Negative	0101	0301	0801	5701	0301	0701	0201	0303
Normalpair2-2	batch4_c7	F	51	Negative	Negative	Negative								
Normalpair3-1	4635311072_B	M	44	Negative	Negative	Negative	0101	1101	0702	3701	0801	1502/1501	0402	0602
Normalpair3-2	4635311043_F	M	44	Negative	Negative	Negative								
Normalpair4-1	block6_a9	F	27	Negative	Negative	Negative	0101	2402	0801	3502/3503	0301	1104	0201	0301
Normalpair4-2	block6_a4	F	27	Negative	Negative	Negative								
Normalpair5-1	block5_b1	M	29	Negative	Negative	Negative	0101	0201/0224	5001	5601	0701	0801	0202	0402
Normalpair5-2	block5_b2	M	29	Negative	Negative	Negative								
Normalpair6-1	batch4_d5	F	30	Negative	Negative	Negative	0301	3201	-	-	0403	1502	0301	0602
Normalpair6-2	batch4_c12	F	31	Negative	Negative	Negative								
Normalpair7-1	block5_b10	F	20	Negative	Negative	Negative	1101	3002	1801	3501/3503	0101	0301	0201	0501
Normalpair7-2	block6_b10	F	20	Negative	Negative	Negative								
Normalpair8-1	4635311075_K	F	33	Negative	Negative	Negative	0101	2920	0801	4403	-	-	0202	0302
Normalpair8-2	4635311075_J	F	33	Negative	Negative	Negative								
Normalpair9-1	4635311084_E	F	18	Negative	Negative	Negative	0101	1101	0705/0706	0801	0701	0701	0202	0303
Normalpair9-2	4635311084_J	F	18	Negative	Negative	Negative								

Pre/post-T1D singletons

Sample ID	Illumina27K array ID	SEX	Disease duration (months)	Age at sampling (years)	GAD	IA2	ICA
1B-Pre	block7_a10	F		18	Positive	Positive	Positive
1B-Post	block7_d12		62		Positive	Positive	Positive
2B-Pre	block7_c11	F		17	Positive	Positive	Positive
2B-Post	block7_d2		27		Positive	Positive	Positive
3B-Pre	block7_a12	M		18	Positive	Positive	Positive
3B-Post	block7_c12		38		Positive	Positive	Positive
4B-Pre	block7_a7	M		16	Positive	Positive	Positive
4B-Post	block7_b12		93		Positive	Positive	Positive
5B-Pre	block7_b11	F		18	Positive	Positive	Positive
5B-Post	block7_d8		42		Positive	Positive	Positive
7B-Pre	block7_b6	F		20	Positive	Positive	Positive
7B-Post	block7_d9		88		Positive	Positive	Positive
9B-Pre	block7_c7	M		18	Positive	Positive	Positive
9B-Post	block7_a5		85		Positive	Positive	Positive
