# Supplementary material for: Identification of Type 1 Diabetes–Associated DNA Methylation Variable Positions That Precede Disease Diagnosis
Source: PLoS Genet. 2011 Sep 29;7(9):e1002300. doi: 10.1371/journal.pgen.1002300 (PMC3183089; doi:10.1371/journal.pgen.1002300)
Supplement: Supplementary file 4 [file pgen.1002300.s005.doc]

CpG ID	Gene name	P-value	T1Dpair1	T1Dpair2	T1Dpair3	T1Dpair4	T1Dpair6	T1Dpair7	T1Dpair8	T1Dpair10	T1Dpair11	T1Dpair12	T1Dpair13	T1Dpair14	T1Dpair15	T1Dpair16	T1Dpair17
cg24041977	*CERK*	0.00012207	-2.9	-4.1	-1.4	-0.6	-0.7	-1.0	-0.5	-0.9	-0.6	-3.1	-0.3	-1.3	-0.1	-1.1	0.0
cg02318426	*NFKBIA*	0.00012207	-0.8	-1.0	-1.4	-1.6	-1.5	0.1	-1.3	-0.5	-0.6	-1.6	-0.4	-1.7	-0.3	-1.6	-1.7
cg17741572	*CFB*	0.000183105	-0.8	-2.4	-0.7	-0.3	-2.4	-1.6	-1.2	0.2	-0.6	-1.4	-2.3	-1.2	-0.3	-0.1	-0.5
cg02941816	*MGMT*	0.000305176	-2.4	-0.7	-1.3	-0.5	0.4	-2.4	-15.2	-2.5	-0.8	-0.5	-0.6	-0.7	-2.3	0.1	-0.9
cg04599297	*GAD2*	0.000427246	-4.2	-5.3	-6.1	-3.1	-1.9	1.5	-7.3	-1.1	-9.7	-6.2	-3.6	-6.6	-3.2	-2.0	0.5
cg04341084	*TPD52*	0.000427246	-0.2	-0.4	-0.3	-1.9	0.2	-0.2	-0.7	-0.1	-0.8	-1.7	-0.8	-1.2	-1.0	-1.1	-0.5
cg08216808	*ARPC2*	0.000610352	-1.3	-1.5	-8.0	1.5	-57.2	-3.3	-3.1	-1.5	-0.3	-7.9	-0.7	-3.4	-8.6	-2.0	-0.3
cg19219366	*TRIM3*	0.000610352	-1.3	-1.3	-1.8	-7.1	-1.3	-4.0	-2.1	-3.5	-2.4	-2.6	0.9	1.0	-3.2	-2.7	-0.9
cg07360076	*FGFR3*	0.000610352	-1.8	-2.0	-0.3	-7.1	-0.7	0.2	0.0	-0.3	-4.0	0.0	-1.7	-1.3	-4.2	-5.3	-2.5
cg09218130	*PUS1*	0.000610352	-1.7	-0.6	-0.8	-1.8	-2.4	0.1	-3.1	-2.9	-0.4	-0.4	-0.2	-1.0	-2.8	-0.6	0.4
cg01889448	*HLA-DQB1*	0.000854492	-2.8	-5.0	-14.0	-1.5	1.2	-5.9	-0.1	-1.5	-1.0	-3.4	0.2	-5.1	-3.6	-8.6	-4.7
cg14480463	*INTS1*	0.000854492	-1.5	-1.5	-5.0	-5.8	-5.2	-8.0	-3.1	-0.1	-3.8	-1.1	0.4	0.5	-0.5	-1.9	-2.9
cg02844629	*ARV1*	0.000854492	-2.7	-1.0	-6.2	-0.8	-3.5	0.7	-1.0	0.1	-6.6	-1.0	0.5	-6.5	-3.6	-1.5	-2.7
cg18075454	*MTRF1L*	0.000854492	0.3	-1.5	-0.9	-2.5	0.1	-0.3	-0.1	-0.2	-1.3	-0.8	-2.7	-0.6	-4.3	-0.5	-1.8
cg17738133	*CDKN1C*	0.000854492	-1.1	-0.3	-1.1	-2.2	-1.2	-1.0	-0.2	0.6	0.0	-0.2	-1.0	-1.5	-0.2	-0.8	-1.0
cg12316147	*GBA2*	0.00115967	-5.6	-3.0	-2.1	-1.5	-1.8	-0.7	-3.4	-0.9	-5.2	-1.6	1.3	-3.6	1.3	-2.0	-2.0
cg17043366	*ZNF414*	0.00115967	-0.5	-0.3	-0.3	-0.2	-0.8	-0.4	0.0	0.3	-0.2	-0.4	-0.7	-0.1	-0.4	-0.6	-0.4
cg10968815	*BPIL1*	0.00152588	-1.8	-3.2	-0.7	-3.1	-1.9	-0.3	-1.1	-2.2	-0.5	-8.0	-2.8	-0.6	0.6	-0.9	0.6
cg08896053	*SNTG1*	0.00152588	-1.3	-0.6	-1.5	-0.3	-3.7	-1.2	-2.6	0.3	-1.7	-3.1	0.1	-2.6	0.4	-1.0	-0.9
cg17567317	*POLR2F*	0.00152588	-0.6	0.1	-0.7	-0.1	-2.2	-0.6	0.3	-0.1	-0.4	-0.2	-0.6	-0.3	-0.7	-1.0	-0.5
cg25060573	*CCND1*	0.00152588	-0.3	-0.1	0.2	-0.9	-0.2	-0.2	-0.3	0.1	-1.0	-0.5	-0.1	-1.4	-0.6	-0.3	-1.1
cg02047577	*UCKL1*	0.00152588	-0.7	-0.3	-0.1	0.0	-0.3	-0.1	0.0	0.1	-0.1	0.0	-0.4	-0.6	-0.1	-0.1	-0.2
cg27546237	*COL4A1*	0.00201416	-2.1	-1.1	-1.2	-7.6	-2.7	-2.8	0.3	-0.3	-1.3	-3.6	-2.4	0.8	0.0	-0.2	-2.4
cg04916311	*PQLC3*	0.00201416	-0.9	-1.2	1.0	-0.9	-0.8	-1.9	0.5	-2.2	-0.8	-0.4	-1.3	-1.0	-1.5	-1.0	-1.2
cg06255524	*DNAJC3*	0.00201416	0.0	-0.6	0.3	-2.1	-1.4	-1.0	-0.6	-0.4	-1.9	0.3	-0.5	-0.9	-0.5	-1.8	0.1
cg16041660	*PRICKLE1*	0.00201416	-0.1	-0.8	-0.8	-0.1	0.2	-0.7	-0.9	-0.5	-2.2	-0.2	-0.6	0.2	-2.3	-0.1	-0.9
cg11510839	*OCA2*	0.00262451	-4.8	-1.1	-2.6	0.7	-1.4	0.8	-2.0	0.5	-2.0	-1.7	0.8	-1.8	-1.3	-2.8	-1.6
cg11999384	*GRN*	0.00262451	-0.8	-1.3	-1.4	0.5	-2.1	-0.3	-1.0	0.9	-0.6	-0.8	-1.5	-4.4	-2.6	0.0	-1.4
cg25013852	*PGM1*	0.00262451	-0.1	-0.1	-0.9	-0.9	-2.4	0.2	0.5	-0.3	-1.3	-1.1	-0.5	-1.3	-1.1	-1.9	-0.4
cg27244432	*ACP6*	0.00262451	-0.7	0.3	-1.0	-0.9	-0.3	-1.6	-0.6	-0.8	-0.8	-0.3	-0.5	0.1	0.5	-1.1	-0.6
cg06588247	*TRAF3*	0.00262451	-0.1	-0.4	-1.0	-0.6	-0.1	-0.2	-0.5	-0.1	0.1	0.1	0.1	-1.2	-0.8	-0.8	-0.3
cg21674595	*C12orf54*	0.00335693	-6.1	-0.9	-1.7	-3.9	-1.3	-11.9	-1.9	5.9	0.3	-8.6	-1.7	-9.0	-9.7	-4.2	-2.5
cg03835292	*LGALS2*	0.00335693	-4.6	-6.3	-3.2	-6.1	-4.5	-6.0	0.5	-7.0	-1.2	2.3	-1.3	-3.6	-5.1	3.0	-2.5

CpG ID	Gene name	P-value	T1Dpair1	T1Dpair2	T1Dpair3	T1Dpair4	T1Dpair6	T1Dpair7	T1Dpair8	T1Dpair10	T1Dpair11	T1Dpair12	T1Dpair13	T1Dpair14	T1Dpair15	T1Dpair16	T1Dpair17
cg04995095	*CD300E*	0.00335693	-2.8	-3.2	0.1	2.3	-3.6	-5.0	-2.7	-1.8	-3.5	-8.9	-3.8	0.0	0.3	-1.5	-4.6
cg15118503	*AHSA2*	0.00335693	-1.4	-3.0	-2.4	-1.2	-3.0	-0.5	-2.0	-3.3	-3.3	-8.1	2.0	-5.9	0.3	0.0	-0.3
cg14108394	*C12orf61*	0.00335693	-1.0	-1.5	-2.0	-1.8	-0.9	-3.8	0.1	2.3	-1.1	-3.3	-0.6	-3.8	-4.7	-2.4	-0.5
cg01282432	*C14orf101*	0.00335693	0.4	-2.1	-2.0	-0.9	-1.9	-1.3	-1.1	-2.1	-3.3	-3.5	-2.4	-5.3	1.0	-2.0	2.0
cg00200063	*USP32*	0.00335693	0.2	-0.6	0.1	0.3	-0.8	-0.2	-0.4	-0.3	-0.6	-0.9	-0.5	0.2	-0.5	-0.7	-1.0
cg15318532	*RFXAP*	0.00335693	-0.2	-0.4	-0.3	-0.7	-0.1	0.2	0.0	-0.6	-0.3	0.1	-0.1	-0.2	-0.6	-0.4	-0.1
cg02104644	*SYT7*	0.00427246	-0.3	-3.7	-1.9	-4.9	-0.8	-1.7	0.9	-0.6	-0.7	-0.7	-4.6	-5.2	-8.5	-2.4	0.8
cg22271212	*TLE2*	0.00427246	-1.9	-0.8	2.6	-5.2	-5.3	-2.5	0.1	-1.4	-0.6	-3.1	-1.3	-0.6	-3.2	-0.6	-1.4
cg04282622	*FAM71C*	0.00537109	-5.2	-2.6	-3.6	8.5	-8.1	-14.7	0.3	-11.7	-3.4	-16.3	-5.6	-17.1	-2.3	-1.5	-0.1
cg10705800	*CITED4*	0.00537109	1.5	0.7	-0.9	-2.0	-1.2	-3.9	-3.2	-1.4	-2.3	-0.7	-0.5	-1.0	-0.1	-1.3	-4.0
cg01775414	*PHF21B*	0.00537109	-2.3	-2.1	-4.1	-3.7	-1.5	0.2	-0.2	1.1	-1.6	-2.4	-0.2	-1.6	1.0	-0.4	-1.8
cg01103730	*IL20*	0.00537109	-1.4	-0.1	-4.5	0.0	0.0	-4.0	-1.2	-0.3	-3.0	-2.6	-2.6	-0.7	0.1	1.8	-0.9
cg19136717	*ARIH1*	0.00537109	-1.1	-2.1	-3.5	-0.3	-4.1	-1.5	0.5	1.5	-0.1	-1.9	-0.5	-0.9	-0.8	-1.1	-3.4
cg05060662	*HIST4H4*	0.00537109	-0.4	-1.8	-0.5	-0.9	0.6	-0.2	-1.0	-0.6	-0.9	-1.0	-1.4	-0.9	-0.2	0.1	0.3
cg10203523	*MRFAP1L1*	0.00537109	0.2	0.0	-0.1	-2.0	-0.9	-0.9	-1.0	-0.4	-1.2	-0.7	-0.6	0.1	-0.9	-0.4	0.3
cg09906488	*CAMK1D*	0.00537109	-0.6	-0.2	-0.8	-2.0	0.5	-0.5	-0.2	-0.1	-0.4	0.1	0.0	-0.6	-0.1	-0.6	-0.3
cg02975259	*NCK1*	0.00537109	-0.2	-0.1	-1.2	0.4	-0.1	-0.4	-0.1	-0.1	-0.7	-0.2	-0.2	-0.1	-0.3	-0.3	-0.4
cg03945021	*ENSG00000224310*	0.00671387	-4.0	3.0	-8.7	0.0	-13.3	-3.1	-0.5	-2.9	-2.6	-3.4	-2.9	1.5	-2.7	-2.4	-2.2
cg16494477	*FGF18*	0.00671387	-0.4	-6.3	-3.1	-5.1	-1.7	-0.5	-0.7	0.0	-0.3	-7.7	0.6	-3.8	-0.9	-4.9	1.6
cg05628549	*PRKCDBP*	0.00671387	1.9	-4.0	-4.7	-5.9	-0.4	-4.8	-1.3	-1.1	-1.0	-6.2	-1.8	1.1	-2.4	-3.4	0.9
cg10082841	*PIP5KL1*	0.00671387	-1.4	0.4	-1.9	-5.6	-1.7	-5.5	-3.5	0.1	1.6	-5.0	0.5	-3.3	-1.9	-0.2	-2.4
cg23563234	*PCDHGB7*	0.00671387	0.0	1.9	-1.4	0.2	-6.1	-1.2	-1.9	-2.7	-0.7	-1.7	-0.2	-0.1	-2.3	-4.2	-0.9
cg09991975	*JOSD1*	0.00671387	-1.6	-1.2	-2.6	1.7	0.3	-1.3	-0.3	-0.1	-1.4	-2.0	-0.7	-2.2	-0.2	-0.9	-2.1
cg24677780	*CA11*	0.00671387	1.3	-0.2	-3.9	-0.4	-0.3	-1.7	-2.3	-0.3	-1.6	-1.2	-1.6	0.3	-0.2	-0.9	-0.5
cg00770871	*TULP3*	0.00671387	-0.8	0.0	-1.2	-1.0	-0.8	-0.4	-0.1	-0.8	0.2	-0.4	-0.3	0.0	0.3	-0.1	-0.3
cg27559343	*SETD3*	0.00671387	-0.2	-0.1	-1.7	-0.7	-0.6	-0.2	1.3	-0.1	-0.8	-0.2	-0.2	-0.3	-0.9	-0.7	0.0
cg16326979	*OXR1*	0.00671387	-0.2	0.0	-0.4	-0.3	-0.7	-0.9	0.9	-0.2	-0.5	-1.3	0.0	-0.2	-0.5	-0.1	-0.2
cg25870420	*ITGA9*	0.00671387	-0.4	-0.8	0.1	-0.4	0.0	-0.4	0.3	-0.5	-0.2	-0.4	0.3	-0.4	-0.7	-1.1	-0.1
cg22941086	*C12orf40*	0.00836182	-4.5	-4.2	-7.1	-7.2	-16.5	-1.9	-5.8	-4.4	1.8	-2.1	-2.3	3.5	-1.3	-3.7	2.5
cg18062196	*DHX40*	0.00836182	-2.4	-2.6	-0.6	-5.2	-11.1	-0.3	1.7	-6.7	0.5	-8.7	-7.4	3.3	-6.1	-0.8	-5.4
cg07310661	*CUL3*	0.00836182	1.6	-0.2	-10.4	-6.1	-5.2	1.1	-0.3	-10.2	3.6	-7.9	-1.7	-7.0	-3.6	-1.7	-2.9
cg00892393	*C16orf73*	0.00836182	3.1	-4.5	-0.3	-6.7	-1.6	-5.4	-4.8	1.0	2.6	-13.3	-0.7	-6.1	-3.8	-2.8	-4.9
cg10692870	*FN1*	0.00836182	-0.9	-0.3	1.0	-7.8	-2.0	-0.8	-2.8	-1.8	0.2	-4.2	-1.1	-1.5	-1.9	1.3	-0.3

CpG ID	Gene name	P-value	T1Dpair1	T1Dpair2	T1Dpair3	T1Dpair4	T1Dpair6	T1Dpair7	T1Dpair8	T1Dpair10	T1Dpair11	T1Dpair12	T1Dpair13	T1Dpair14	T1Dpair15	T1Dpair16	T1Dpair17
cg21885650	*TBCCD1*	0.00836182	-0.8	1.6	-4.5	-1.0	-1.8	-0.6	-0.6	-3.4	-1.1	-1.0	-0.3	0.5	-0.5	-0.6	-1.2
cg04807655	*TIAM1*	0.00836182	0.2	0.9	-1.7	0.0	-2.2	-1.3	0.9	-0.6	-3.3	-2.4	-0.3	-1.1	-0.6	-1.8	-1.2
cg01936124	*ETF1*	0.00836182	-0.1	-0.2	-1.6	-1.5	0.2	-2.3	0.3	0.3	-1.8	-0.7	0.0	-1.1	-1.2	-1.8	-1.7
cg11638880	*SFT2D1*	0.00836182	-1.2	-0.3	-1.4	0.3	-1.2	-1.3	0.7	0.5	-0.5	-0.7	0.2	-2.1	-1.9	-1.2	-1.4
cg15786541	*PVRL3*	0.00836182	0.1	-0.6	-0.7	-1.4	0.0	0.3	-0.3	0.1	-0.1	-0.1	-0.4	-2.4	-0.9	-1.8	-1.3
cg24817430	*MESDC1*	0.00836182	-0.4	1.5	-0.5	-1.8	0.0	-0.4	-0.1	-0.1	-0.6	-0.8	-0.5	-0.8	-0.4	-0.3	-0.4
cg25047102	*VKORC1*	0.00836182	-0.1	0.1	0.2	-0.5	-0.4	-0.4	0.0	0.0	-0.1	0.0	-0.3	0.0	-0.6	-0.3	-0.2
cg19379303	*RNASEH2C*	0.00836182	-0.3	0.0	0.0	-0.6	-0.3	-0.2	0.2	0.0	-0.1	-0.1	-0.1	0.0	-0.1	-0.3	-0.2
cg23957424	*TRAF6*	0.000183105	-0.2	0.8	2.7	0.7	1.2	2.1	0.6	0.8	1.4	0.5	1.9	0.6	2.2	0.0	0.5
cg25406518	*DAK*	0.000183105	0.9	2.7	8.3	3.4	1.2	2.7	2.4	5.0	-0.1	1.6	1.1	3.8	0.0	6.3	3.4
cg11484872	*TNF*	0.000305176	1.4	4.5	5.9	8.2	2.7	2.6	2.7	3.9	6.1	2.4	-1.0	2.4	0.7	0.7	3.6
cg09516965	*PTGDR*	0.000427246	0.3	0.7	0.6	0.9	2.0	-0.4	0.3	0.6	0.9	0.0	0.6	0.8	0.5	1.4	0.9
cg17607231	*SP140*	0.000854492	-0.9	1.9	1.3	1.0	1.9	3.8	-0.6	1.4	2.1	2.1	0.9	2.7	2.3	1.6	0.5
cg23101680	*SPERT*	0.000854492	8.5	3.5	13.5	10.6	6.8	7.0	1.2	-3.4	0.4	4.2	4.8	-0.1	3.8	0.9	3.7
cg24139421	*SLC22A18*	0.00115967	0.5	-0.4	0.0	0.4	0.4	1.4	1.2	0.2	1.2	1.3	1.0	0.4	0.4	0.4	0.0
cg01753595	*COL5A1*	0.00115967	0.7	2.2	0.8	0.8	1.6	0.6	1.4	-0.1	0.0	0.9	-0.5	0.8	0.6	1.1	0.1
cg02515725	*PDLIM3*	0.00115967	-0.3	7.5	2.2	1.0	2.6	0.8	2.8	1.9	1.6	0.4	0.5	1.2	0.2	2.4	-0.8
cg22341310	*ZNF541*	0.00115967	7.5	13.7	3.1	40.3	5.2	-1.9	4.6	2.6	0.5	3.9	0.1	6.0	11.0	-0.6	3.0
cg13843603	*PSMD11*	0.00152588	0.1	1.1	0.2	0.5	-0.2	0.8	0.3	0.2	0.5	0.1	0.9	0.2	0.0	0.5	0.0
cg04951204	*PPP1R9A*	0.00152588	1.2	0.1	1.8	1.1	1.0	-0.2	0.4	0.8	1.4	-0.3	0.8	1.2	0.2	0.0	1.7
cg21516384	*RNF213*	0.00152588	0.2	-0.1	0.1	4.7	1.1	0.7	0.1	2.6	2.1	-0.2	2.2	0.3	0.8	-0.1	0.6
cg14453641	*AGL*	0.00152588	-0.4	1.4	2.0	0.5	0.4	1.5	1.1	0.8	1.0	2.0	3.8	0.8	2.2	0.3	-0.7
cg19691267	*ACYP1*	0.00152588	3.3	0.4	-0.8	1.3	2.5	3.3	1.3	-1.2	3.3	1.7	0.5	1.6	1.1	1.2	2.3
cg24120841	*THRB*	0.00152588	2.1	2.6	-1.0	1.2	0.8	0.0	0.7	5.0	7.4	0.9	2.0	1.9	-0.3	1.1	4.0
cg23074747	*C9orf72*	0.00201416	0.5	0.7	0.0	1.1	1.7	0.0	0.4	0.1	1.1	0.0	-0.4	0.1	1.6	0.4	1.3
cg20339230	*ST8SIA2*	0.00262451	-0.2	1.0	0.3	0.5	0.1	1.4	0.4	0.4	0.4	0.6	0.0	-0.1	0.2	0.0	0.5
cg08466883	*KCTD3*	0.00335693	0.4	1.4	1.1	0.5	-0.2	0.2	0.3	0.0	0.9	-0.1	0.8	-0.2	0.4	0.2	0.6
cg04167054	*ATP2C1*	0.00335693	0.4	0.2	1.0	0.1	-0.3	1.9	0.4	0.0	1.4	0.5	0.5	0.3	0.4	0.2	-0.2
cg23154002	*PATZ1*	0.00335693	1.1	0.0	2.5	0.8	0.9	0.7	0.3	0.2	-0.9	0.9	0.2	1.3	0.8	1.4	0.0
cg22474507	*CINP*	0.00427246	0.5	1.2	0.9	0.4	0.3	0.1	0.6	0.0	1.6	1.4	0.9	0.1	-1.2	0.0	0.0
cg19287114	*SLC44A1*	0.00427246	0.6	0.8	0.7	1.7	1.0	-0.2	0.1	-0.1	0.7	-0.3	0.3	0.3	1.5	2.4	0.2
cg07915542	*IDH1*	0.00427246	1.1	3.1	1.8	6.2	1.2	1.5	0.9	1.0	-3.2	0.3	1.3	3.4	3.2	0.2	3.4
cg09551147	*SORCS3*	0.00537109	0.1	0.3	0.3	0.1	0.4	0.1	0.0	0.2	0.8	0.0	1.3	0.7	-0.5	0.5	0.2

CpG ID	Gene name	P-value	T1Dpair1	T1Dpair2	T1Dpair3	T1Dpair4	T1Dpair6	T1Dpair7	T1Dpair8	T1Dpair10	T1Dpair11	T1Dpair12	T1Dpair13	T1Dpair14	T1Dpair15	T1Dpair16	T1Dpair17
cg05111829	*SUPV3L1*	0.00537109	0.1	0.2	1.0	-0.1	0.3	0.2	0.2	-0.4	0.1	2.7	0.5	0.2	0.1	0.3	0.1
cg09902130	*CD6*	0.00537109	0.4	0.2	0.8	0.2	0.5	0.9	-0.5	-0.4	1.7	0.8	0.3	0.7	0.3	0.7	1.1
cg13982661	*SC5DL*	0.00537109	1.1	-0.2	1.9	0.7	0.5	0.0	-0.1	1.8	-0.5	0.5	1.3	0.6	0.0	0.4	0.5
cg23442323	*CD109*	0.00537109	1.0	-0.1	2.3	0.4	0.3	0.0	0.8	0.2	0.4	0.6	2.7	2.1	-0.4	-0.2	0.8
cg04398978	*CBX4*	0.00537109	4.7	0.5	6.5	5.4	2.9	3.0	1.6	5.8	2.7	4.6	0.9	4.4	-2.8	4.0	-3.3
cg25791888	*HSF2BP*	0.00671387	0.3	1.2	0.5	0.8	0.3	-0.2	0.2	1.1	0.5	-0.5	0.2	0.9	0.0	0.6	0.5
cg09539408	*STRN3*	0.00671387	1.1	0.4	0.9	0.5	0.7	1.2	0.0	0.0	0.1	0.6	1.5	-0.9	0.0	0.0	0.0
cg10185638	*ATG9A*	0.00671387	0.3	0.1	1.9	0.8	0.1	0.5	0.0	0.1	0.1	0.5	2.1	0.8	-0.6	0.6	-0.1
cg09648233	*ALKBH4*	0.00671387	-0.2	-0.2	1.9	0.6	1.5	1.1	0.4	0.0	0.9	1.0	0.6	0.3	-0.1	0.8	0.0
cg02735071	*EIF3H*	0.00671387	0.3	0.5	2.3	1.0	0.4	0.8	0.1	1.1	0.4	0.9	1.1	0.4	0.4	0.1	-1.2
cg09848198	*EEA1*	0.00671387	0.8	0.4	-0.2	1.9	0.6	3.1	0.2	-0.7	0.7	-0.2	1.2	0.7	1.1	0.8	0.1
cg05637351	*CDH7*	0.00671387	0.8	0.8	1.4	0.4	0.7	-1.3	2.6	1.9	0.8	1.2	1.9	0.8	1.7	-0.4	-0.3
cg14013300	*XBP1*	0.00671387	1.6	1.7	2.4	0.8	1.2	0.9	1.0	1.9	0.0	3.6	-0.6	1.0	-1.0	0.2	0.5
cg06379754	*CACNA2D1*	0.00671387	2.2	0.9	0.4	3.4	0.6	1.9	2.3	0.5	0.4	1.6	0.2	3.0	-2.1	0.2	-0.2
cg27437944	*FXR1*	0.00671387	0.6	1.4	4.4	0.7	0.8	0.8	-0.5	1.3	-0.2	1.7	2.3	2.0	0.9	-0.9	0.0
cg01437411	*ATN1*	0.00671387	0.9	2.4	-0.1	4.3	-0.7	0.3	1.0	0.8	1.9	0.5	2.8	1.8	0.2	-0.7	0.5
cg20509080	*ARHGEF3*	0.00671387	0.4	0.7	0.6	0.6	4.8	2.3	-2.5	0.4	2.6	-0.1	0.5	1.6	2.4	2.4	1.0
cg23299127	*MAP3K6*	0.00671387	2.8	0.8	2.2	0.6	-1.2	-0.1	1.7	0.3	-1.6	2.3	3.2	2.2	0.1	3.6	2.9
cg11277230	*C4orf36*	0.00671387	-0.1	-0.3	0.9	0.3	1.7	-0.6	0.4	2.3	3.7	7.4	4.7	7.6	2.0	2.2	-0.7
cg11318251	*BAALC*	0.00671387	1.6	3.8	2.1	8.7	-0.1	0.6	-0.4	2.6	0.6	7.3	2.9	5.5	1.0	-3.5	1.9
cg24365013	*CLRN1*	0.00671387	7.3	1.4	19.0	15.3	-0.6	-0.4	10.1	0.8	2.7	1.6	1.3	2.3	2.8	2.4	-3.3
cg09339301	*QKI*	0.00836182	0.4	0.3	-0.4	0.1	2.7	0.4	0.0	0.2	0.8	-0.2	0.0	0.6	0.1	1.0	0.4
cg18633178	*C16orf80*	0.00836182	1.5	-0.1	0.0	0.3	1.5	1.1	0.2	-0.7	0.8	0.4	0.0	0.4	0.9	0.8	0.4
cg18473162	*ZNF787*	0.00836182	0.6	0.0	0.6	0.5	0.6	0.9	0.7	0.4	-0.3	1.2	2.5	-0.9	0.5	0.6	0.0
cg17808849	*HERPUD1*	0.00836182	0.3	0.5	1.9	-0.7	1.3	-0.1	1.1	1.3	0.8	0.9	2.3	1.0	0.0	0.1	-0.5
cg02181494	*ARSA*	0.00836182	0.3	2.1	0.7	2.5	0.6	-0.8	0.4	0.6	0.7	0.1	0.0	1.3	1.2	1.3	-0.5
cg18769818	*ARAP1*	0.00836182	0.1	1.3	-0.2	1.7	0.5	1.2	0.8	-0.2	0.6	-0.4	-0.2	0.2	1.6	2.9	1.7
cg18704595	*ADA*	0.00836182	0.5	1.3	0.9	2.2	1.2	2.9	2.0	-1.2	0.4	3.2	0.5	1.2	0.1	-0.4	-0.3
cg21850069	*TRIM41*	0.00836182	0.2	-0.1	2.2	3.4	1.0	4.9	-1.1	0.1	0.2	1.0	0.1	2.9	1.4	-0.1	0.2
cg21413009	*DAPK2*	0.00836182	1.2	0.3	3.9	1.4	1.6	-0.1	1.2	0.7	1.4	3.7	4.6	2.2	-1.9	1.3	-0.3
cg11903057	*RHOH*	0.00836182	-1.2	1.4	3.8	4.1	2.0	3.6	-0.4	1.6	0.8	3.5	3.5	0.4	-0.1	1.6	-1.1
cg11086066	*NFATC2*	0.00836182	1.4	0.9	1.1	2.6	10.2	7.0	0.2	-0.8	1.5	-1.6	-0.3	1.4	1.1	4.1	2.4
cg16992787	*CAPS*	0.00836182	-3.3	2.5	3.0	3.6	3.7	-0.3	2.5	-0.1	0.8	8.3	1.2	0.2	2.0	7.4	2.0
